# Comparative Performance of 3 Analytical Models in Identifying Associated Factors of Pulmonary Dysfunction–Depression Comorbidity: China Health and Retirement Longitudinal Study–Based Nationwide Cross-Sectional Study

**DOI:** 10.2196/77940

**Published:** 2026-04-09

**Authors:** Qinglin Cheng, Qiancheng Cao, Weilin Teng, Ruoqi Dai, Qingjun Jia, Xuexin Bai, Qingchun Li, Yifei Wu, Yinyan Huang

**Affiliations:** 1 Department of Tuberculosis Control and Prevention, Hangzhou Center for Disease Control and Prevention (Hangzhou Health Supervision Institution), Mingshi 568#, Shangcheng District, Hangzhou, 310021, Zhejiang, China, +86-571-85155039, +86-571-85177371; 2 School of Public Health and Nursing, Hangzhou Normal University, Hangzhou, China

**Keywords:** depression screening, pulmonary dysfunction, comorbidity, analytical models, Bayesian networks

## Abstract

**Background:**

Pulmonary dysfunctions are common and frequently co-occur with depressive symptoms, worsening outcomes, and increasing health care burden. Clinically usable models for identifying pulmonary dysfunction–depression comorbidity remain limited by suboptimal interpretability, inconsistent validation, and uncertain generalizability.

**Objective:**

This study developed and compared logistic regression (LR), Bayesian network (BN), and Extreme Gradient Boosting (XGBoost) models for identifying factors associated with pulmonary dysfunction–depression comorbidity and evaluated their clinical usefulness across different decision thresholds.

**Methods:**

Data were drawn from the 2011 and 2015 waves of the China Health and Retirement Longitudinal Study. The analytical sample comprised 1146 adults with confirmed pulmonary dysfunction, of whom 514 (44.9%) exhibited clinically significant depressive symptoms (10-item Center for Epidemiologic Studies Depression Scale [CESD-10] score of ≥10). Models incorporated demographic, biomarker, comorbidity, and behavioral variables. Performance was assessed via discrimination (area under the receiver operating characteristic curve [AUROC]), calibration (Hosmer-Lemeshow test), and decision curve analysis. Sensitivity analyses excluding psychiatric history addressed potential conceptual overlap with the outcome.

**Results:**

LR and BN showed similar discrimination across cohorts (AUROC≈0.73), exceeding XGBoost (0.690 training; 0.650 validation). LR had the most balanced validation performance (specificity 0.721; sensitivity 0.647), whereas BN favored sensitivity (0.884) over specificity (0.401). Training calibration was good for LR or BN, but only LR remained acceptable in validation; XGBoost was miscalibrated. XGBoost’s training net benefit did not generalize. Psychiatric history was the strongest factor (odds ratio 3.46‐7.63), followed by nephropathy, arthritis, and gastropathy; BMI and household registration were inversely associated. Excluding psychiatric history modestly reduced AUROC. With 20 shared predictors, AUROCs converged (0.658‐0.665), BN calibrated best, LR or BN remained sensitivity-forward, and XGBoost remained specificity-forward.

**Conclusions:**

Using routinely available clinical and sociodemographic variables, LR and BN matched or exceeded XGBoost in externally validated performance and produced more reliable probability estimates. Model choice should align with intended use: BN (or LR) is preferable for sensitivity-forward screening, whereas XGBoost may be reserved for high-threshold confirmatory decisions only after recalibration. Across methods, psychiatric history, nephropathy, arthritis, gastropathy, household registration status, and BMI emerged as stable markers of vulnerability to depressive symptoms in pulmonary dysfunction.

## Introduction

Pulmonary dysfunctions (PDs) are defined as a pathological state characterized by impaired ventilation (obstructive or restrictive patterns) and/or gas exchange abnormalities (diffusion limitation and ventilation-perfusion mismatch), leading to hypoxemia and/or hypercapnia [1,2]. Common etiologies include chronic obstructive pulmonary disease (COPD), asthma, interstitial lung disease, pulmonary fibrosis, and pulmonary embolism [1,2]. Pulmonary dysfunction–depression comorbidity (PDDC) constitutes a clinically significant syndemic, jointly contributing to accelerated morbidity, mortality, and increased health care burdens globally [3]. PDs affect more than 500 million individuals worldwide and are frequently complicated by depressive disorders, a comorbidity associated with 23%‐40% reductions in treatment adherence and a 1.8-fold increase in hospitalization rates [4-6]. The bidirectional relationship between PDs and depression has been extensively documented, with accumulating evidence demonstrating their synergistic impact on clinical outcomes. For instance, individuals with PDs, particularly pulmonary tuberculosis, COPD, and cystic fibrosis, exhibit a 1.5‐ to 2.5-fold higher risk of developing depressive symptoms than the general population [7]. While the prevalence of depression in the general Chinese population is approximately 10.6% [8], it reaches up to 39.5% among patients with particularly pulmonary tuberculosis [9]. A matched cohort study further reported that the incidence of depression was 11.4 per 1000 person-years among patients with COPD, compared with 5.7 per 1000 person-years in non-COPD controls [10].

Longitudinal analyses confirm the progressive influence of depressive symptomatology on pulmonary deterioration [11,12]. Multivariable models further demonstrated that depression severity independently predicted reduced forced expiratory volume in 1 second as a percentage of the predicted value (FEV1%) at 2-year follow-up [11]. Conversely, impaired lung function has also been found to independently predict the onset of depression, potentially mediated by hypoxemia, systemic inflammation, and glucocorticoid dysregulation [12]. The underlying pathophysiological mechanisms involve neuroendocrine-immune cross talk, including proinflammatory cytokine release (eg,interleukin-6 and tumor necrosis factor–α) induced by chronic hypoxia and hypercapnia, which disrupt monoamine neurotransmission and hippocampal neurogenesis—hallmarks of depression [13,14]. Socioeconomic disparities further exacerbate these effects, with stronger associations observed between lung function decline and subsequent depression in populations of lower socioeconomic status [15]. Despite growing understanding of this bidirectional relationship, several critical gaps remain. First, most studies use cross-sectional designs, limiting causal inference regarding temporal dynamics. Second, although pulmonary rehabilitation and selective serotonin reuptake inhibitors have demonstrated efficacy in improving both psychological and pulmonary outcomes (eg, FEV1), the biological pathways mediating these dual benefits remain poorly characterized [16]. Third, standardized screening protocols for PDDC are inconsistently applied, resulting in underdiagnosis and undertreatment.

Clinically, the prognostic implications of this comorbidity are substantial. A German cohort study of patients with cystic fibrosis revealed that depressive symptoms interacted with baseline FEV1% to predict disease trajectories: individuals with preserved lung function (FEV1>70%) but comorbid depression experienced significantly steeper declines (ΔFEV1=–5.2% vs –1.3% in nondepressed counterparts over 2 years) [11]. These findings emphasize the importance of early psychological screening within pulmonary care frameworks. However, current predictive models remain insufficiently integrated and fail to capture the full complexity of this syndemic.

Traditional statistical methods, such as logistic regression (LR), have been widely used in early research due to their transparency and interpretability. Nevertheless, these approaches have notable limitations: univariate selection strategies may overlook important interaction effects (eg, the combined influence of biomarkers and psychosocial stress), and linear assumptions may be inadequate for modeling threshold phenomena in disease progression (eg, a sharp decline in lung function triggering psychological decompensation) [17,18]. In parallel, machine learning techniques—such as random forests, Bayesian networks (BNs), and Extreme Gradient Boosting (XGBoost)—have gained increasing traction in medical prediction [19]. These algorithms offer theoretical advantages in identifying complex patterns through automated feature engineering and nonparametric modeling. However, existing applications often neglect 3 key challenges: limited clinical interpretability (black box models hinder mechanistic understanding), sample size dependency (complex algorithms may perform suboptimally in small datasets), and insufficient validation strategies (lack of robust assessment across time and geographic regions).

Despite expanding literature, 3 unresolved issues persist. First, methodological constraints remain: current models prioritize predictive accuracy over clinical interpretability, thereby limiting their use in guiding mechanistic insights or informing therapeutic decisions. Second, generalizability remains questionable, as many studies rely on single-center or region-specific data, undermining external validity across diverse populations. Third, nonlinear relationships and synergistic interactions—such as FEV1 thresholds precipitating depression onset or the combined influence of biological and psychosocial factors—are inadequately addressed.

To address these limitations, the study pursues 3 objectives. First, model development: we propose a hybrid analytical framework that integrates traditional statistical methods (eg, LR), explainable machine learning (eg, BNs), and advanced machine learning algorithms (eg, XGBoost). This framework is designed to balance predictive performance with clinical interpretability, thereby supporting actionable decision-making. Second, framework exploration: BN analysis will be used to identify key mediators—such as biomarkers, sociodemographic characteristics, and comorbidity indicators—and to clarify their conditional dependencies within PDDC. Third, validation strategy: model performance will be rigorously assessed using bootstrap resampling and DeLong tests to evaluate discrimination across spatial and temporal dimensions while addressing sample size constraints.

Through these aims, the study seeks to develop a clinically actionable model for PDDC that can inform personalized screening and targeted interventions, with the potential to reduce the burden of this syndemic. Specifically, we aim to (1) compare the discriminative performance and clinical operating characteristics of LR, BN, and XGBoost models; (2) identify core factors consistently associated with PDDC across analytical approaches; and (3) assess the clinical usefulness of each model for screening versus confirmatory diagnostic applications. We hypothesize that although machine learning models may better capture complex nonlinear relationships, traditional statistical methods will achieve comparable discrimination with greater interpretability, and that chronic multimorbidity patterns and sociodemographic factors will emerge as dominant correlates across all modeling frameworks.

## Methods

### Data Source and Inclusion Criteria

The study used data from the 2011 and 2015 waves of the China Health and Retirement Longitudinal Study (CHARLS) [20], a nationally representative, longitudinal cohort study organized by the National School of Development at Peking University. The CHARLS database uses a multistage, stratified probability sampling design, spanning 28 provinces, municipalities, and autonomous regions, and encompasses 150 counties. It contains comprehensive health-related data for more than 20,000 individuals aged 45 years and older, including standardized assessments of demographic characteristics, health status and functional capacity, health care utilization and insurance coverage, and socioeconomic status, collected via structured questionnaires, clinical examinations, and laboratory assays.

**Figure 1.**
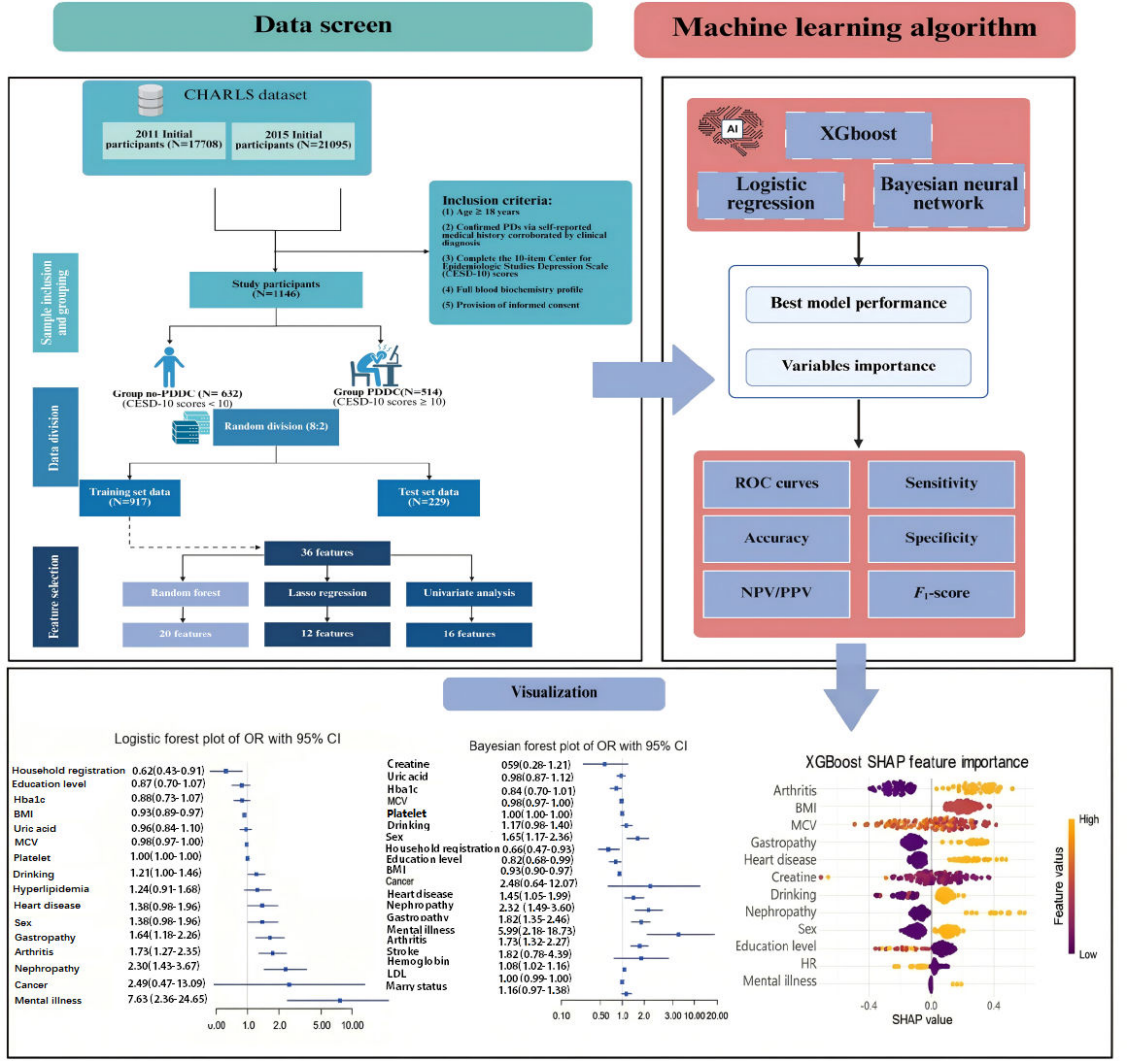
This flowchart outlines the machine learning analysis pipeline based on the CHARLS dataset. AI: artificial intelligence; CESD-10: Center for Epidemiologic Studies Depression Scale-10; CHARLS: China Health and Retirement Longitudinal Study; HbA_1c_: hemoglobin A_1c_; HR: household registration; LDL: low-density lipoprotein; MCV: mean corpuscular volume; NPV/PPV: negative/positive predictive value; OR: odds ratios; PDs: pulmonary dysfunctions; PDDC: pulmonary dysfunction–depression comorbidity; ROC: receiver operating characteristic; SHAP: Shapley Additive Explanations; XGBoost: Extreme Gradient Boosting.

Participants were selected through a rigorous multistage process. This study workflow was summarized in Figure 1. In the initial screening phase, all individuals with self-reported lung disease diagnoses or respiratory symptoms were identified from the database. From 38,803 CHARLS participants in the 2011 and 2015 waves, we applied sequential inclusion criteria: (1) age ≥18 years, (2) confirmed PD, (3) complete depression assessment, and (4) <20% missing predictor data. PD was defined using a composite criterion incorporating both self-reported physician diagnosis of chronic lung diseases (chronic bronchitis, emphysema, asthma, or pulmonary heart disease; corresponding to ICD-10 [*International Statistical Classification of Diseases, Tenth Revision*] codes J40-J47) and, where available, spirometry-confirmed airflow limitation (postbronchodilator FEV₁/FVC <0.70 per Global Initiative for Chronic Obstructive Lung Disease criteria) [21]. This dual approach minimizes misclassification bias inherent in single-source definitions [22]. Exclusions included severe cognitive impairment (Mini-Mental State Examination <18) and terminal malignancies. The final analytical sample comprised 1146 participants.

### Variables and Data Collection

This analytical framework used multidimensional variables systematically categorized into four distinct domains: (1) Demographic parameters including age, sex, BMI, education level, household registration, and marital status. (2) Blood-based biomarkers comprising glucose, cholesterol, high-density lipoprotein, low-density lipoprotein, triglycerides, blood urea nitrogen, creatine, uric acid, high-sensitivity C-reactive protein, hemoglobin A_1c_ (HbA_1c_), hematocrit, hemoglobin, white blood cell, mean corpuscular volume (MCV), and platelet. (3) Chronic disease comorbidity quantified through binary classification (yes/no) of 13 clinically significant noncommunicable conditions: hypertension, diabetes, cancer, hyperlipidemia, hepatopathy, heart disease, stroke, nephropathy, gastropathy, mental illness (defined as physician-diagnosed depression, anxiety, or other psychiatric conditions documented in medical history, distinct from current depressive symptoms assessed by 10-item Center for Epidemiologic Studies Depression Scale [CESD-10]), memory-associated disorders, arthritis, and asthma. Notably, while “mental illness” as a predictor and CESD-10–defined depression as an outcome are related constructs, they capture different dimensions: the former reflects historical clinical diagnosis, whereas the latter measures current symptom severity. (4) Behavioral determinants encompassing smoking and drinking. More details are reported in Table S1 in Multimedia Appendix 1.

### Outcome Specification

Depressive symptomatology was assessed using the CESD-10 [23], a psychometrically validated screening instrument in population-based studies. Participants rated symptom frequency over the preceding week (eg, depressed mood, loneliness, and fear) on a 4-point Likert scale (total score range: 0‐30). Consistent with established epidemiological thresholds, scores ≥10 were classified as clinically significant depressive symptoms, demonstrating robust sensitivity (91.93%) and specificity (92.76%) in prior validation studies [23].

### Statistical Analysis

This study used a cross-sectional design using pooled data from 2 CHARLS waves (2011 and 2015) to develop and validate an analytical framework for identifying factors associated with prevalent depressive symptoms in patients with PDs. These waves were selected because they contain the most complete blood biomarker panels, which were essential for our multidimensional analytical approach. More recent CHARLS waves (2018 and 2020) have incomplete biomarker data due to changes in data collection protocols and COVID-19 pandemic disruptions. The pooled analytical sample comprised 1146 participants. The dataset was partitioned through random allocation, with 80% (917) of participants constituting the derivation cohort for model development, while the remaining 20% (229) served as an independent validation cohort to assess model generalizability.

### Model Construction

#### Data Preprocessing

Systematic preprocessing was performed prior to model development. Missing values for continuous variables were imputed using multiple imputation techniques, while missing categorical variables were coded as a distinct category to retain maximal information.

#### LR Model Construction

We constructed a conventional LR model as a reference comparator, adopting a hierarchical analytical approach to variable selection. The methodology proceeded through two sequential phases: (1) Bonferroni-adjusted univariate screening (α≤.05) to identify candidate associated factors exhibiting preliminary associations with incident PDDC, followed by (2) an iterative refinement process using forward stepwise regression (entry threshold: α<.05) to achieve optimal model parsimony while preserving discriminative capacity. The final LR model took the form:


$$\log\left(\frac{p}{1-p}\right)=\beta_{0}+\sum_{i=1}^{k}\beta_{i}X_{i}$$

where *p* denotes the probability of depressive symptoms, *β*₀ the intercept, and *βᵢ* the coefficients for predictors *Xᵢ*.

#### BNs Model Construction

We implemented a BN model using the R package brms (BN Models using Stan, version 2.18.0). The modeling procedure comprised three principal components: (1) Associated-factor selection: twenty clinically relevant variables were identified through random forest–based feature importance analysis, spanning demographic parameters, blood-based biomarkers, chronic disease comorbidity, and behavioral determinants. (2) Prior distribution specification: weakly informative normal priors (N(0, 1)) were assigned to all regression coefficients, providing sufficient regularization while maintaining theoretical flexibility [24]. (3) Markov Chain Monte Carlo sampling configuration: the model used 4 parallel Markov Chain Monte Carlo chains, each executing 2000 iterations (1000 warm-up and 1000 sampling iterations) [25]. Convergence was optimized by setting the adaptation parameter (adapt_delta) to 0.95, effectively reducing divergent transitions while maintaining sampling efficiency.

### XGBoost Model Construction

The XGBoost model construction used a 2-stage pipeline integrating Least Absolute Shrinkage and Selection Operator (LASSO) regularization for feature selection followed by gradient boosting for classification [26].

#### Feature Selection via LASSO

LASSO regression with L1 penalty was applied to all candidate variables. The optimal regularization coefficient (λ) was determined via 10-fold cross-validation, selecting the λ value within 1 standard error of the minimum cross-validated deviance (λ=0.023). This procedure yielded 12 variables with nonzero coefficients: mental illness, nephropathy, arthritis, gastropathy, household registration, BMI, sex, heart disease, drinking, education level, creatinine, and MCV. The relatively stringent feature selection reflects LASSO’s tendency to select 1 representative variable among correlated predictors, which may have excluded potentially informative features.

#### Hyperparameter Optimization

XGBoost hyperparameters were optimized via grid search with 5-fold cross-validation on the training set. The parameter grid included max_depth (3, 4, 5, and 6), learning rate η (0.01, 0.05, 0.1, and 0.2), n_estimators (100, 200, and 300), subsample (0.7, 0.8, and 0.9), and colsample_bytree (0.7, 0.8, and 0.9). The optimal configuration maximizing cross-validated area under the receiver operating characteristic curve (AUROC) was max_depth=6, *η*=0.1, n_estimators=200, subsample=0.8, and colsample_bytree=0.8. Early stopping was implemented with a patience of 20 rounds to prevent overfitting. The objective function minimized:


$$L(\theta )=\sum_{i=1}^{n}l\;\left(y_{i},{\hat{y}}_{i}\right)\;+\;\lambda \sum_{j=1}^{m}|w_{j}|$$

where *l*(*yᵢ, ŷᵢ*) denotes the logistic loss, *wⱼ* the feature weights, and *λ* the regularization strength.

### Model Validation

All models were validated using a comprehensive set of metrics: (1) Primary metric: AUROC, with 95% CIs computed via the DeLong method. (2) Secondary metrics: accuracy, sensitivity, specificity, positive predictive value, and negative predictive value, each reported with point estimates and 95% CI. (3) Calibration: model calibration was assessed using calibration curves plotting observed versus predicted probabilities across deciles of predicted risk, with Hosmer-Lemeshow goodness-of-fit test statistics. (4) Clinical usefulness: decision curve analysis (DCA) was performed to evaluate net benefit across a range of clinically relevant threshold probabilities (0.1‐0.6), comparing each model with “treat all” and “treat none” strategies [27].

To facilitate fair model comparison, we additionally conducted a unified feature set analysis in which all 3 models were fit using the same prespecified predictor set (defined a priori based on clinical relevance and data availability). We reconstructed all 3 models using an identical 20-feature (ie, household registration, sex, education level, BMI, marital status, creatine, HbA_1c_, MCV, platelet, LDL, uric acid, hemoglobin, cancer, nephropathy, heart disease, gastropathy, arthritis, mental illness, stroke, and drinking) input set. Performance metrics (discrimination, calibration, and decision-curve net benefit) were then compared under identical inputs. All analyses were conducted in R (version 4.4.3) using validated computational libraries (brms, rstan, dplyr, pROC, caret, xgboost, rmda, ggplot2, and forestplot), with complete reproducibility scripts archived in Table S2 in Multimedia Appendix 1.

### Ethical Considerations

This study adhered to the principles of the Declaration of Helsinki and received ethical approval from the Biomedical Ethics Committee of Peking University (approval no. IRB00001052-11015). The requirement for informed consent was waived by the ethics committee because the study was based on a secondary analysis of routinely collected administrative data and did not involve any direct contact with individual participants. Both the original data collection and the current secondary analysis were approved without the need for additional informed consent. All data from the CHARLS were anonymized before analysis. Identifiable information had been removed by the data custodian, and the authors had no access to any personal information that could enable the identification of individual participants. Data access and analytical procedures were carried out in accordance with applicable data protection requirements. No financial or other incentives were provided to participants, as the study did not involve direct enrollment of or interaction with human subjects. Neither the manuscript nor the supplementary materials contains any images or other information that could reveal the identity of individual participants.

## Results

### Baseline Characteristics

The study cohort comprised 1146 participants (non-PDDC: n=632; PDDC: n=514), with baseline characteristics detailed in Table S3 in Multimedia Appendix 1. Demographic comparisons revealed no significant age difference between groups (mean 62.48, SD 9.93 vs mean 62.16, SD 9.67 years; *P*=.58), although marked disparities emerged in BMI (mean 23.20, SD 4.22 vs mean 22.54, SD 3.71 kg/m²; *P*=.01), sex distribution (male: 372/632, 58.9% vs 232/514, 45.1%; *P*<.001), educational attainment (high school or above: 169/632, 26.7% vs 65/514, 12.6%; *P*<.001), and urban household registration (215/632, 34.0% vs 100/514, 19.5%; *P*<.001). Marital status showed no statistical significance (*P*=.08). Hematologic profiling identified elevated uric acid (mean 4.81, SD 1.37 vs mean 4.43, SD 1.20 mg/dL; *P*<.001) and HbA_1c_ (mean 5.3, SD 1% vs mean 5.2, SD 1%; *P*=.002) in the non-PDDC group, alongside increased MCV (mean 91.59, SD 8.47 vs mean 90.25, SD 8.91 fL; *P*=.01) and reduced platelet counts (mean 194, SD 95 vs mean 202, SD 93×10⁹/L; *P*=.05). No significant differences were observed in glucose, lipid profiles, renal markers, or inflammatory indices. Chronic comorbidity analysis demonstrated substantially lower prevalence rates in the non-PDDC group for cancer (3/632, 0.5% vs 10/514, 1.9%; *P*=.02), hyperlipidemia (281/632, 44.5% vs 281/514, 54.7%; *P*=.001), heart disease (107/632, 16.9% vs 150/514, 29.2%; *P*<.001), and organ-specific pathologies (nephropathy: 46/632, 7.3% vs 88/514, 17.1%, *P*<.001; gastropathy: 130/632, 20.6% vs 211/514, 41.1%, *P*<.001). Behavioral assessments revealed differential alcohol consumption patterns (*P*<.001), with increased drinking frequency (≥ monthly: 171/632, 27.1% vs 98/514, 19.1%) and reduced nondrinkers (404/32, 64.0% vs 386/514, 75.1%) among patients with non-PDDC.

### Nonunified Feature Set Analysis

#### Modeling Construction

##### LR Analysis

**Figure 2.**
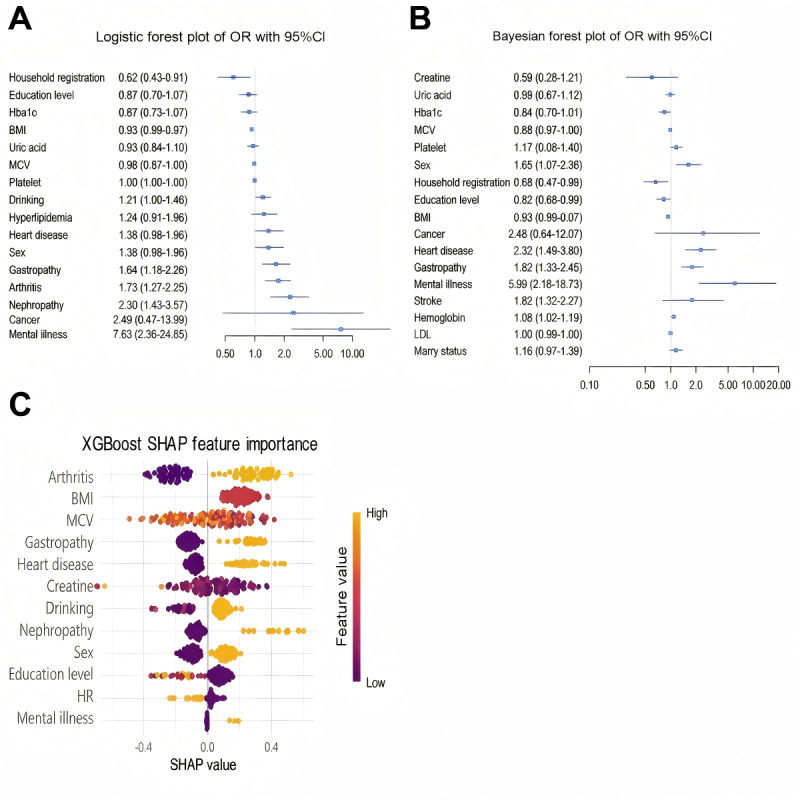
Visualization analyses of the 3 models in the nonunified feature set. (**A**) Logistic regression model, (**B**) Bayesian network model, and (**C**) Extreme Gradient Boosting model. HbA_1c_: hemoglobin A_1c_; HR: household registration; LDL: low-density lipoprotein; MCV: mean corpuscular volume; OR: odds ratios; SHAP: Shapley Additive Explanations.

Sixteen significant associated factors (*P*<.05) were identified through univariate analysis, encompassing sociodemographic factors (BMI, sex, education level, and household registration), blood-based biomarkers (uric acid, HbA_1c_, MCV, and platelet), chronic disease comorbidity (cancer, hyperlipidemia, heart disease, nephropathy, arthritis, gastropathy, and mental illness), and behavioral determinant (drinking) in the training dataset (Table 1). Multivariable LR analyses identified mental illness as the most robust associated factors of PDDC (odds ratios [ORs] 7.63, 95% CI 2.36‐24.65), with subsequent clinical correlates demonstrating descending effect magnitudes: nephropathy (OR 2.30, 95% CI 1.43‐3.67), arthritis (OR 1.73, 95% CI 1.27‐2.35), gastropathy (OR 1.64, 95% CI 1.18‐2.26), and drinking (OR 1.21, 95% CI 1.00‐1.46). Notably, inverse associations emerged for household registration status (OR 0.62, 95% CI 0.43‐0.91) and BMI (OR 0.93, 95% CI 0.89‐0.97), suggesting potential protective effects (Figure 2A).

**Table 1.** Univariate analysis of the training dataset and validation dataset.

Variables^a^^,b^	Training dataset (N=917)			Validation dataset (N=229)		
	Group no-PDDC^c^ (n=496)	Group PDDC (n=421)	*P* value	Group no-PDDC (n=136)	Group PDDC (n=93)	*P* value
Demographic parameters						
Age (years), mean (SD)	62.60 (10.07)	62.01 (9.72)	.37	62.07 (9.45)	62.83 (9.49)	.55
BMI, (kg/m²), mean (SD)	23.29 (4.37)	22.53 (3.81)	.01	22.85 (3.60)	22.61 (3.26)	.40
Sex, n (%)			<.001			.05
Male	294 (59.27)	192 (45.61)		78 (57.35)	40 (43.01)	
Female	202 (40.73)	229 (54.39)		58 (42.65)	53 (56.99)	
Education level, n (%)			<.001			.08
Primary school or below	290 (58.47)	328 (77.91)		78 (57.35	66 (70.97)	
Middle school	70 (14.11)	46 (10.93)		25 (18.38)	9 (9.68)	
High school or above	136 (27.42)	47 (11.16)		33 (24.26)	18 (19.35)	
Household registration, n (%)			<.001			.66
Rural area	327 (65.93)	349 (82.90)		90 (66.18)	65 (69.89)	
Urban area	169 (34.07)	72 (17.10)		46 (33.82)	28 (30.11)	
Marital status, n (%)			.05			.25
Married	432 (87.10)	348 (82.66)		116 (85.29)	74 (79.57)	
Separated and divorced	1 (0.20)	7 (1.66)		2 (1.47)	0 (0.00)	
Widowed	59 (11.90)	60 (14.25)		18 (13.24)	18 (19.35)	
Never married	4 (0.81)	6 (1.43)		0 (0.00)	1 (1.08)	
Blood-based biomarkers, mean (SD)						
Glucose (mg/dL)	106.40 (31.95)	104.71 (23.58)	.36	104.87 (27.93)	103.09 (21.11)	.58
Cholesterol (mg/dL)	190.55 (38.10)	189.67 (35.84)	.72	189.92 (36.36)	191.01 (39.90)	.83
HDL^d^ (mg/dL)	53.67 (16.70)	53.22 (15.89)	.68	51.45 (14.54)	54.50 (13.72)	.11
LDL^e^ (g/dL)	111.66 (33.08)	113.14 (29.84)	.48	112.33 (32.24)	112.58 (32.64)	.95
TG^f^ (mg/dL)	122.83 (70.62)	122.26 (68.34)	.90	127.22 (78.07)	117.37 (67.64)	.31
BUN^g^ (mg/dL)	16.09 (4.93)	15.69 (4.84)	.22	15.57 (4.35)	16.52 (5.07)	.14
Creatine (mg/dL)	0.84 (0.26)	0.78 (0.21)	<.001	0.79 (0.18)	0.76 (0.17)	.22
Uric acid (mg/dL)	4.83 (1.42)	4.46 (1.21)	<.001	4.71 (1.19)	4.29 (1.17)	.01
hsCRP^h^ (mg/dL)	3.43 (9.10)	3.59 (8.03)	.77	4.39 (11.19)	2.64 (5.34)	.12
HbA_1c_^i^ (%)	5.44 (87.00)	5.26 (66.00)	<.001	5.44 (80.00)	5.39 (70.00)	.61
Hematocrit (mg/dL)	41.83 (6.04)	41.54 (6.06)	.47	42.67 (5.28)	41.41 (5.35)	.08
Hemoglobin (mg/dL)	14.27 (2.03)	14.42 (2.27)	.32	14.64 (1.99)	14.37 (1.73)	.27
WBC^j^ (10^9^/L)	6.30 (2.26)	6.49 (2.33)	.20	6.33 (2.04)	6.29 (1.94)	.88
MCV^k^ (fl)	91.41 (8.47)	90.48 (8.62)	.10	92.21 (8.46)	89.23 (10.12)	.02
Platelet (10^9^/L)	201.32 (70.56)	211.70 (76.98)	.04	199.64 (70.83)	207.94 (70.53)	.38
Chronic disease comorbidity, n (%)						
Hypertension			.96			.70
No	327 (65.93)	276 (65.56)		86 (63.24)	62 (66.67)	
Yes	169 (34.07)	145 (34.44)		50 (36.76)	31 (33.33)	
Diabetes			.46			.90
No	428 (86.29)	355 (84.32)		119 (87.50)	80 (86.02)	
Yes	68 (13.71)	66 (15.68)		17 (12.50)	13 (13.98)	
Cancer			.03			.99
No	494 (99.60)	412 (97.86)		135 (99.26)	92 (98.92)	
Yes	2 (0.40)	9 (2.14)		1 (0.74)	1 (1.08)	
Hyperlipidemia			.004			.12
No	274 (55.24)	191 (45.37)		77 (56.62)	42 (45.16)	
Yes	222 (44.76)	230 (54.63)		59 (43.38)	51 (54.84)	
Hepatopathy			.47			.98
No	461 (92.94)	385( 91.45)		130 (95.59)	88 (94.62)	
Yes	35 (7.06)	36 (8.55)		6 (4.41)	5 (5.38)	
Heart disease			<.001			.05
No	411 (82.86)	297 (70.55)		114 (83.82)	67 (72.04)	
Yes	85 (17.14)	124 (29.45)		22 (16.18)	26 (27.96)	
Stroke			.23			.41
No	484 (97.58)	404 (95.96)		136 (100.00)	92 (98.92)	
Yes	12 (2.42)	17 (4.04)		0 (0.00)	1 (1.08)	
Nephropathy			<.001			.04
No	457 (92.14)	346 (82.19)		129 (94.85)	80 (86.02)	
Yes	39 (7.86)	75 (17.81)		7 (5.15)	13 (13.98)	
Gastropathy			<.001			.02
No	396 (79.84)	245 (58.19)		106 (77.94)	58 (62.37)	
Yes	100 (20.16)	176 (41.81)		30 (22.06)	35 (37.63)	
Mental illness			.01			.16
No	491 (98.99)	404 (96.00)		135 (99.26)	89 (95.70)	
Yes	5 (1.01)	17 (4.00)		1 (0.74)	4 (4.30)	
Memory-associated disorders			.41			.99
No	488 (98.39)	410 (97.39)		132 (97.06)	90 (96.77)	
Yes	8 (1.61)	11 (2.61)		4 (2.94)	3 (3.23)	
Arthritis			<.001			.16
No	341 (68.75)	185 (43.94)		84 (61.76)	48 (51.61)	
Yes	155 (31.25)	236 (56.06)		52 (38.24)	45 (48.39)	
Asthma			.23			.24
No	409 (82.46)	333 (79.10)		111 (81.62)	69 (74.19)	
Yes	87 (17.54)	88 (20.90)		25 (18.38)	24 (25.81)	
Behavioral determinants, n (%)						
Smoking			.87			.34
Nonsmoker	216 (43.55)	181 (42.99)		64 (47.06)	37 (39.78)	
Smoker	280 (56.45)	240 (57.01)		72 (52.94)	56 (60.22)	
Drinking			<.001			.40
Drink more than once a month	138 (27.82)	82 (19.48)		33 (24.26)	16 (17.20)	
Drink but less than once a month	46 (9.27)	24 (5.70)		10 (7.35)	6 (6.45)	
None of these	312 (62.90)	315 (74.82)		93 (68.38)	71 (76.34)	

a Continuous variables are presented as mean (SD). Normality was assessed using the Shapiro-Wilk test. Variables meeting normality assumptions (BMI, glucose, HDL, LDL, MCV, and hemoglobin) were compared using Student *t* test. Nonnormally distributed variables (TG, BUN, creatinine, uric acid, hsCRP, HbA_1c_, hematocrit, WBC, and platelet) were compared using the Mann-Whitney *U* test.

b Categorical variables are presented as n (%) and compared using Pearson chi-square test or Fisher exact test (for expected cell counts <5).

c PDDC: pulmonary dysfunction–depression comorbidity.

d HDL: high-density lipoprotein.

e LDL: low-density lipoprotein.

f TG: triglycerides.

g BUN: blood urea nitrogen.

h hsCRP: high-sensitivity C-reactive protein.

i HbA_1c_: hemoglobin A_1c_.

j WBC: white blood cell.

k MCV: mean corpuscular volume.

##### BN Analysis

The BN model was constructed through 2 methodologically integrated phases. First, variable selection was conducted via random forest feature importance analysis, which identified 20 clinically significant associated factors across four domains: (1) demographic characteristics (household registration, sex, education level, BMI, and marital status), (2) hematologic biomarkers (creatine, HbA_1c_, MCV, platelet, LDL, uric acid, and hemoglobin), (3) chronic comorbidities (cancer, nephropathy, heart disease, gastropathy, arthritis, mental illness, and stroke), and (4) behavioral determinants (drinking; Figure S1 in Multimedia Appendix 1). These variables demonstrated substantial analytical capacity. Second, the final model with Bayesian forest plot verification demonstrated mental illness as the strongest associated factor (OR 5.99, 95% CI 2.18‐18.73), followed by nephropathy (OR 2.32, 95% CI 1.49‐3.60), gastropathy (OR 1.82, 95% CI 1.35‐2.46), arthritis (OR 1.73, 95% CI 1.32‐2.27), sex (OR 1.65, 95% CI 1.17‐2.36), heart disease (OR 1.45, 95% CI 1.05‐1.99), and hemoglobin (OR 1.08, 95% CI 1.02‐1.16). Protective associations were identified for household registration status (OR 0.66, 95% CI 0.47‐0.93), education level (OR 0.82, 95% CI 0.68‐0.99), and BMI (OR 0.93, 95% CI 0.90‐0.97) (Figure 2B).

##### XGBoost Model Analysis

LASSO regression selected 12 robust associated factors, with 6 variables (mental illness, nephropathy, arthritis, gastropathy, household registration, and BMI; 50%) overlapping across all models. Arthritis, mental illness, gastropathy, sex, heart disease, nephropathy, and drinking were consistently identified as positive associated factors, whereas BMI, household registration status, education level, creatine, and MCV demonstrated inverse associations (Figure S2 in Multimedia Appendix 1). Feature importance analysis via Shapley Additive Explanations (SHAP) values identified arthritis (mean |SHAP|=0.409), BMI (mean |SHAP|=0.376), and MCV (mean |SHAP|=0.350) as the 3 most salient associated factors of PDDC, with hierarchical dominance persisting across all model configurations (Figure 2C).

### Sensitivity Analysis Excluding Mental Illness

To mitigate conceptual overlap between physician-diagnosed psychiatric history (“mental illness”) and the CESD-10 outcome, we repeated the analyses after removing this covariate from all 3 models. In the LR model, nephropathy (OR 1.94, 95% CI 1.20‐3.14), arthritis (OR 1.85, 95% CI 1.35‐2.53), gastropathy (OR 1.93, 95% CI 1.38‐2.70), household registration status (OR 0.67, 95% CI 0.46‐0.98), and BMI (OR 0.92, 95% CI 0.88‐0.96) remained significantly associated with the outcome. Similar associations were observed in the BN model: nephropathy (OR 2.10, 95% CI 1.30‐3.54), arthritis (OR 1.95, 95% CI 1.40‐2.74), gastropathy (OR 1.84, 95% CI 1.33‐2.54), household registration status (OR 0.62, 95% CI 0.43‐0.84), and BMI (OR 0.92, 95% CI 0.87‐0.94). In the XGBoost model, the following features were identified as important contributors based on their SHAP values: arthritis (SHAP=0.260), gastropathy (SHAP=0.202), education level (SHAP=0.144), sex (SHAP=0.108), BMI (SHAP=0.106), household registration status (SHAP=0.079), MCV (SHAP=0.073), and creatine (SHAP=0.073). Model discrimination declined modestly (BN AUROC: 0.735-0.698, LR AUROC: 0.734-0.698, and XGBoost AUROC: 0.690-0.677) but remained acceptable, indicating that predictive performance was not primarily driven by the psychiatric history variable. The consistent, limited attenuation across models supports the independent predictive contribution of the remaining factors.

### Discrimination, Calibration, and Classification Performance

**Figure 3.**
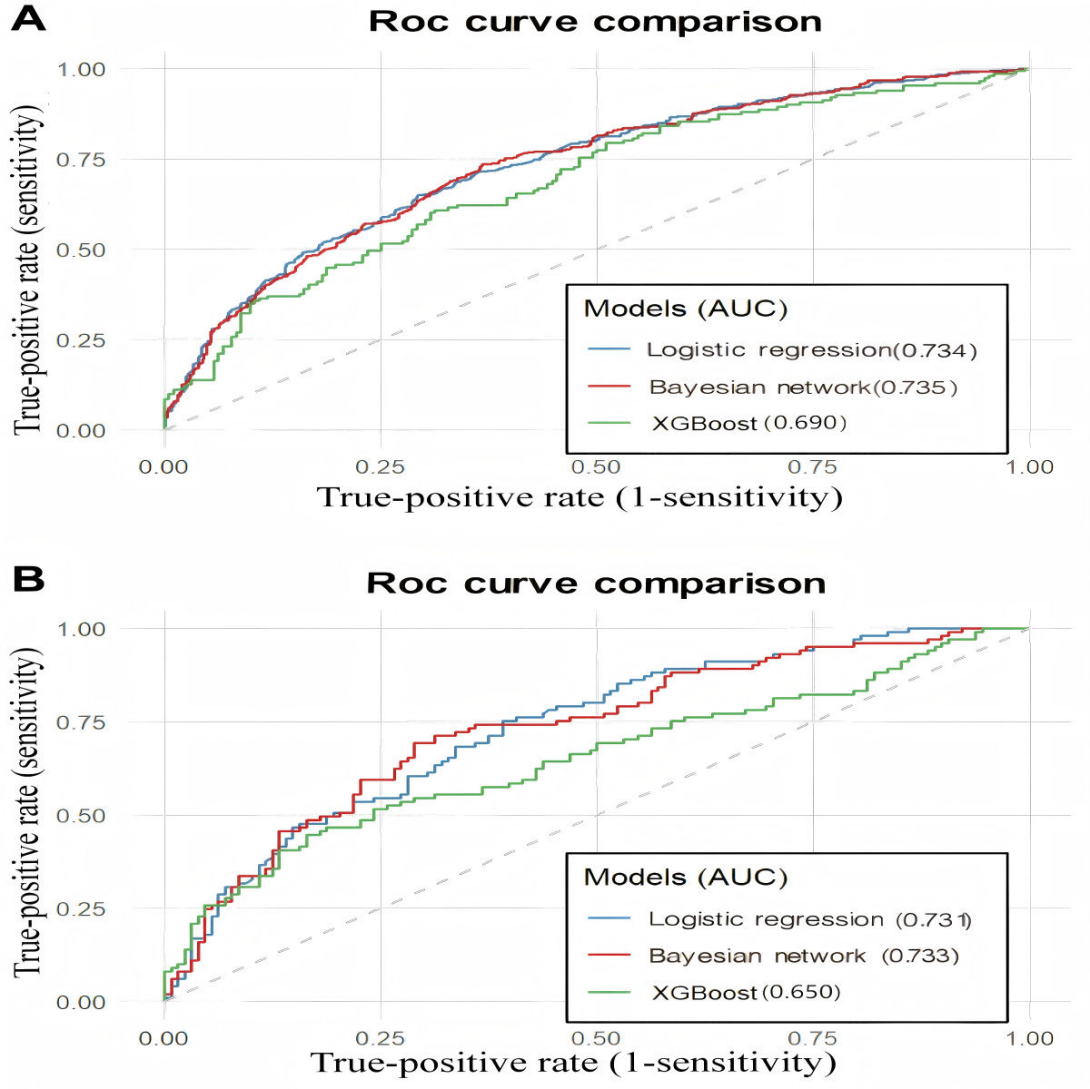
Comparative performance of the 3 models in analyzing pulmonary dysfunction–depression comorbidity through receiver operating characteristic analysis in the nonunified feature set. (**A**) Discriminative capacity of logistic regression, Bayesian network, and Extreme Gradient Boosting models in the training dataset. (**B**) External validation of model generalizability in a test dataset. ROC: receiver operating characteristic; XGBoost: Extreme Gradient Boosting.

To evaluate the discriminative capacity of analytical models in analyzing PDDC, receiver operating characteristic analysis was performed on both training and validation cohorts (Table 2 and Figure 3). In the training dataset, the LR model achieved an AUROC of 0.734 (95% CI 0.708‐0.752) with balanced performance metrics (sensitivity 0.633 and specificity 0.735). The BN demonstrated comparable discrimination (AUROC 0.735, 95% CI 0.709‐0.760) but showed distinct operational characteristics (sensitivity 0.894 vs specificity 0.389). The XGBoost algorithm yielded relatively lower predictive accuracy (AUROC 0.690, 95% CI 0.638‐0.731) with moderate sensitivity (0.558) and specificity (0.444; Figure 3A). This performance pattern persisted in the test set, where LR maintained robust discrimination (AUROC 0.731, 95% CI 0.700‐0.759) with preserved sensitivity (0.647) and specificity (0.721). BN showed similar AUROC performance (AUROC 0.733, 95% CI 0.709‐0.770) but continued to exhibit high sensitivity (0.884) at the expense of specificity (0.401). XGBoost demonstrated reduced generalizability (AUROC 0.650, 95% CI 0.603‐0.688) with suboptimal operational characteristics (Figure 3B; Table S4 in Multimedia Appendix 1).

**Table 2.** Comparison of discrimination metrics across 3 models.

Metrics	Bayesian network model				Logistic regression model				Extreme Gradient Boosting model			
	Training set ^a^		Test set ^b^		Training set ^a^		Test set ^b^		Training set ^a^		Test set ^b^	
Threshold	0.612	0.527	0.591	0.375	0.407	0.493	0.364	0.373	0.504	0.468	0.515	0.509
Sensitivity	0.894	0.548	0.884	0.755	0.633	0.597	0.647	0.755	0.558	0.697	0.553	0.510
Specificity	0.389	0.814	0.401	0.635	0.735	0.763	0.721	0.635	0.444	0.792	0.561	0.833
Accuracy	0.668	0.695	0.664	0.689	0.679	0.688	0.682	0.689	0.499	0.750	0.491	0.689
Positive predictive value	0.643	0.706	0.621	0.626	0.746	0.672	0.731	0.626	0.482	0.732	0.503	0.712
Negative predictive value	0.749	0.689	0.773	0.762	0.620	0.699	0.687	0.762	0.520	0.762	0.517	0.677
F1.F1	0.748	0.618	0.651	0.684	0.685	0.632	0.665	0.684	0.517	0.714	0.437	0.594
Area under the receiver operating characteristic curve	0.735	0.749	0.733	0.661	0.734	0.753	0.731	0.665	0.690	0.844	0.650	0.658

a The first and second columns present the metrics for the nonunified and unified feature sets in the training set, respectively.

b The first and second columns present the metrics for the nonunified and unified feature sets in the test set, respectively.

**Figure 4.**
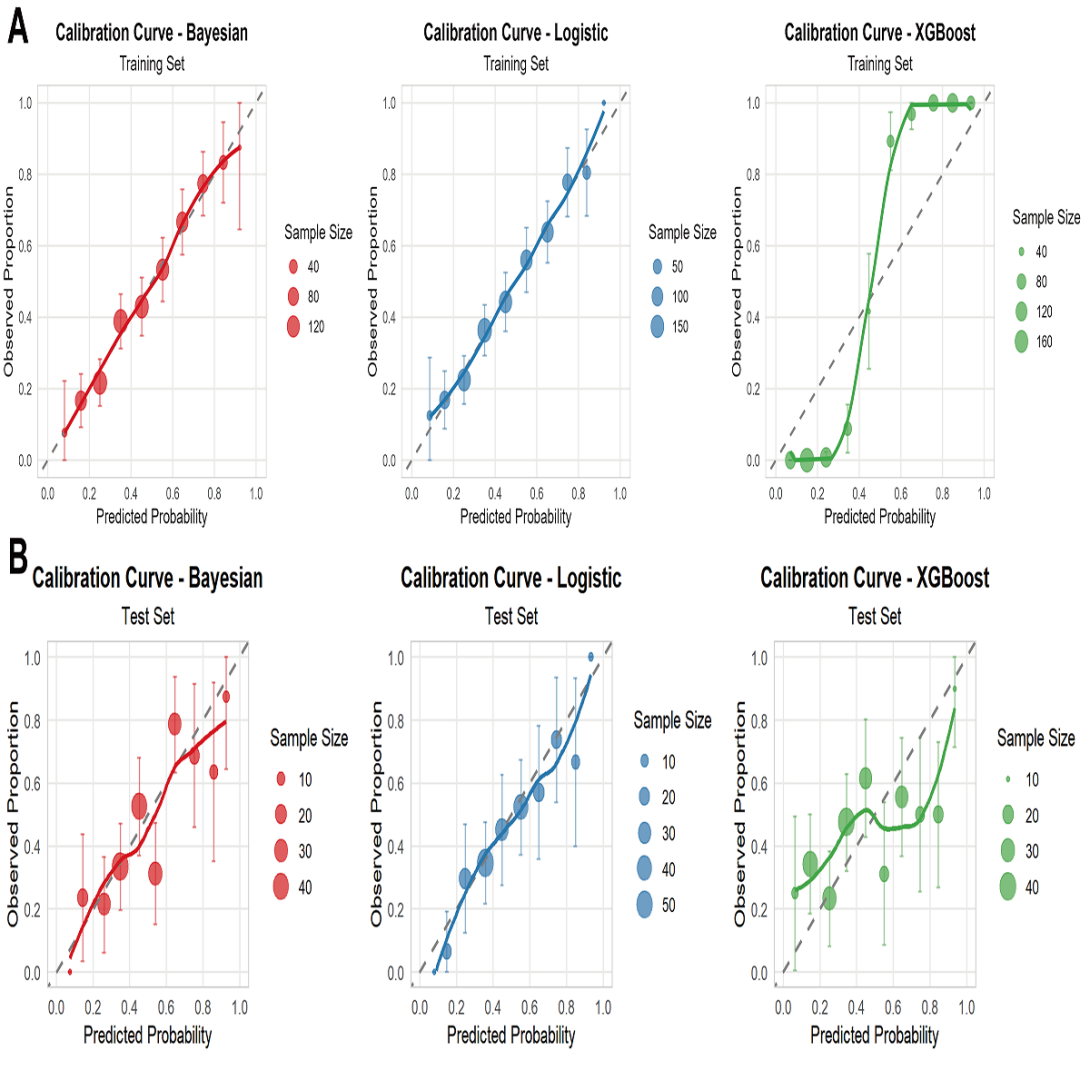
Calibration curve analyses for logistic regression, Bayesian network, and Extreme Gradient Boosting models in analyzing pulmonary dysfunction–depression comorbidity in the nonunified feature set. (**A**) Training set. (**B**) Test set. XGBoost: Extreme Gradient Boosting.

Calibration analyses for all 3 models are shown in Figure 4. In the training set, both LR and BN models demonstrated excellent calibration, with predicted probabilities closely aligned with observed outcomes (Hosmer-Lemeshow test: *P*=.98 and *P*=.99, respectively). In contrast, the XGBoost model exhibited significant miscalibration (*P*<.001), indicating overfitting (Figure 4A). In the test set, only the LR model maintained acceptable calibration (Hosmer-Lemeshow *χ*²_8_=10.71; *P*=.22), although moderate underestimation was observed in lower probability ranges (calibration slope=0.918). The BN model showed significant miscalibration (*χ*²_8_=21.18, *P*=.01; calibration slope=0.858), suggesting limited generalizability. The XGBoost model demonstrated the poorest calibration, with severe deviation across the probability spectrum (calibration slope=0.374; *χ*²_8_=48.93; *P*<.001; Figure 4B). Brier scores in the test set were 0.211 for LR, 0.213 for BN, and 0.256 for XGBoost, indicating comparable overall prediction accuracy between LR and BN. These findings suggest that while XGBoost achieves superior discrimination in the training set, its probability estimates require recalibration before clinical application. The LR model provides more reliable probability estimates with better preserved calibration upon external validation.

**Figure 5.**
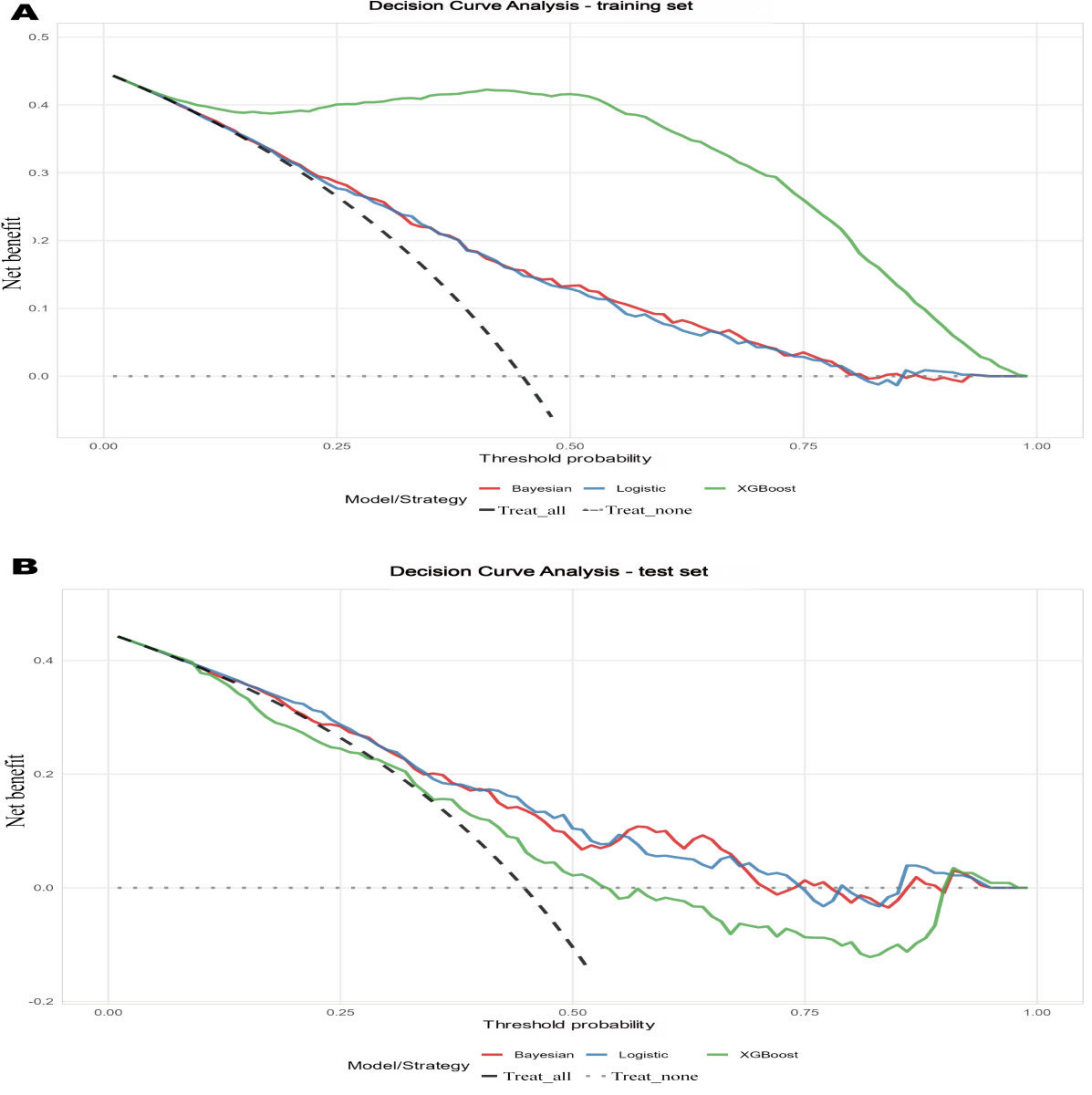
Decision curve analyses for logistic regression, Bayesian network, and Extreme Gradient Boosting models in analyzing pulmonary dysfunction–depression comorbidity in the nonunified feature set. (**A**) Training set. (**B**) Test set. XGBoost: Extreme Gradient Boosting.

DCA revealed distinct threshold-dependent patterns between the training and test sets (Figure 5). In the training set, XGBoost demonstrated the highest net benefit across all threshold probabilities, outperforming both BN and LR models (Figure 5A). This pattern reversed in the test set, where XGBoost yielded lower net benefit than BN and LR across the entire threshold range, indicating limited generalizability (Figure 5B). Within the test set, BN and LR models provided more consistent clinical usefulness. At moderate-to-high thresholds (55%‐60%), the BN model showed marginally higher net benefit than LR. Overall, while XGBoost exhibited superior training set performance, and BN and LR achieved more stable net benefit upon external validation, consistent with overfitting by XGBoost.

### Unified Feature Set Analysis

#### Modeling Construction

##### LR Analysis

In multivariable LR, physician-diagnosed mental illness showed the strongest association with the outcome (OR 4.70, 95% CI 1.51‐14.62). Other significant correlates, in descending magnitude, were nephropathy (OR 2.12, 95% CI 1.31‐3.42), arthritis (OR 1.94, 95% CI 1.42‐2.65), gastropathy (OR 1.84, 95% CI 1.32‐2.58), male sex (OR 1.71, 95% CI 1.17‐2.51), heart disease (OR 1.55, 95% CI 1.07‐2.22), and hemoglobin (OR 1.10, 95% CI 1.02‐1.20). Household registration status (OR 0.61, 95% CI 0.42‐0.90) and BMI (OR 0.93, 95% CI 0.89‐0.96) were inversely associated with the outcome.

##### BN Analysis

The BN model yielded a similar pattern. Mental illness remained the strongest predictor (OR 3.46, 95% CI 1.38‐8.94), followed by nephropathy (OR 2.14, 95% CI 1.32‐3.56), arthritis (OR 1.95, 95% CI 1.42‐2.72), gastropathy (OR 1.86, 95% CI 1.35‐2.56), male sex (OR 1.72, 95% CI 1.19‐2.46), heart disease (OR 1.54, 95% CI 1.05‐2.25), and hemoglobin (OR 1.11, 95% CI 1.02‐1.20). Household registration status (OR 0.61, 95% CI 0.42‐0.86) and BMI (OR 0.92, 95% CI 0.89‐0.96) again showed inverse associations.

##### XGBoost Model Analysis

SHAP analyses from the XGBoost model ranked arthritis as the most influential feature (mean |SHAP| 0.260), followed by gastropathy (0.202), education level (0.144), sex (0.108), and BMI (0.106). These features contributed the largest marginal effects on predictions and accounted for most of the model’s explanatory signal (Figure S3 in Multimedia Appendix 1).

### Sensitivity Analysis Excluding Mental Illness

To reduce potential conceptual overlap between physician-diagnosed psychiatric history (“mental illness”) and the CESD-10 outcome, we repeated the unified feature set analyses after removing this covariate from all 3 models. Model discrimination remained largely unchanged, indicating that predictive performance was robust to this specification. The modest, consistent attenuation observed across models suggests that the remaining multimorbidity and sociodemographic predictors contribute independent prognostic information. Although mental illness was strongly associated with the outcome, the retained covariates continued to provide substantial predictive value (Table S5 in Multimedia Appendix 1).

### Discrimination, Calibration, and Classification Performance

In the training set, LR achieved an AUROC of 0.753, with specificity of 0.763 and sensitivity of 0.597. The BN showed a similar AUROC (0.749) but a distinct operating profile, with higher specificity (0.814) and lower sensitivity (0.548). XGBoost demonstrated superior discrimination (AUROC 0.844), with sensitivity of 0.697 and specificity of 0.792. In the independent test cohort, discriminative performance was comparable across models: LR yielded an AUROC of 0.665 (95% CI 0.595‐0.736), BN 0.661 (95% CI 0.590‐0.731), and XGBoost of 0.658 (95% CI 0.585‐0.731), with no significant pairwise differences (all *P*>.37). Despite similar AUROCs, threshold-dependent performance differed substantially. At their respective optimal thresholds, BN and LR favored sensitivity (0.755 for both), whereas XGBoost prioritized specificity (0.833; Table 2; Figure S4 and Table S4 in Multimedia Appendix 1).

Calibration results are shown in Figure S5 in Multimedia Appendix 1. In the training set, LR and BN exhibited near-perfect calibration (Hosmer-Lemeshow *P*=.84 and *P*=.93, respectively), whereas XGBoost was significantly miscalibrated (*P*<.001), consistent with overfitting. In the test set, only BN retained acceptable calibration (Hosmer-Lemeshow *χ*²_8_=14.23; *P*=.12), despite mild underestimation at lower probabilities (calibration slope=0.645). LR showed moderate miscalibration (*P*=.01; slope=0.613), and XGBoost performed worst, with systematic deviation across the probability range (slope=0.680) and unreliable Hosmer–Lemeshow statistics owing to sparse bins. Brier scores in the test set were 0.227 for XGBoost, 0.229 for LR, and 0.229 for BN, indicating marginally superior point prediction accuracy for XGBoost despite its calibration shortcomings. A composite calibration score integrating Brier score, calibration slope, and Hosmer-Lemeshow results ranked BN highest (0.493), followed by LR (0.182); XGBoost could not be fully scored owing to unreliable statistics. DCA revealed threshold-dependent net benefit (Figure S6 Multimedia Appendix 1). Between 0% and −40% risk threshold, LR and BN outperformed XGBoost, reflecting their higher sensitivity for ruling out disease in screening settings. Above −40%, XGBoost delivered the greatest net benefit, consistent with its high-specificity profile for confirmatory testing (except a narrow range near 60%, where LR was marginally superior). In summary, with identical features, the simpler LR and BN models matched XGBoost’s discriminative performance while offering substantially better-calibrated probabilities. BN provided the most reliable risk estimates overall. For low-risk-threshold (screening) applications, BN or LR is preferred; for high-risk-threshold (confirmatory) decisions, XGBoost can be considered if its probabilities are recalibrated.

## Discussion

### Principal Findings

In a cohort of 1146 participants, PDDC was characterized by a higher burden of chronic comorbidities and less favorable sociodemographic profiles, while most routine laboratory markers showed limited separation between groups. Across modeling strategies, discrimination for PDDC risk was modest and broadly comparable, whereas calibration and threshold-specific operating behavior differed meaningfully. A small set of recurrent factors—psychiatric history, nephropathy, arthritis, gastropathy, household registration status, and BMI—emerged consistently across models and remained informative when psychiatric history was excluded to reduce conceptual overlap with the CESD-10 outcome.

### Impact of Feature Selection

Different feature selection strategies (univariate screening for LR, random forest importance for BN, and LASSO- or SHAP-guided selection for XGBoost) yielded overlapping predictors, suggesting a stable signal rather than method-specific artifacts [28]. Notably, the unified feature set analysis narrowed performance differences and highlighted that model behavior was driven less by discrimination than by calibration and operating thresholds. This finding is practically important: when models are restricted to the same routinely available inputs, simpler approaches can match the discrimination of more complex learners, while often offering more interpretable structure and, in some evaluations, more reliable probabilities.

### Comparative Performance of Analytical Models

#### Discriminative Capacity

In the development data, LR and BN achieved similar discrimination (AUROC=0.73) and consistently outperformed XGBoost (AUROC 0.69 in training, declining to 0.65 in validation), suggesting weaker generalizability for the more complex learner under the original specification. When evaluated in an independent test cohort with identical predictors, all 3 models converged to nearly identical AUROCs (0.66) and did not differ statistically. This convergence indicates that, with the available feature set, algorithmic complexity alone did not translate into improved discrimination; instead, the attainable signal for separating PDDC from non-PDDC appears constrained by the information content of routinely collected variables [29].

#### Clinical Operational Characteristics

Despite similar AUROCs, the models adopted distinct operating points at their optimized thresholds. LR tended to preserve a more balanced sensitivity-specificity trade-off in the development or validation evaluation, whereas BN consistently favored sensitivity at the expense of specificity. This high-sensitivity profile may be advantageous in screening contexts where the primary objective is to minimize missed cases [30]. In contrast, XGBoost favored specificity in the independent test cohort, aligning better with confirmatory strategies where avoiding false positives is prioritized.

DCA underscored that clinical usefulness was threshold dependent and cohort sensitive [31]. In the development set, XGBoost showed higher net benefit across thresholds, but this advantage did not generalize; in the test set, LR and BN provided more stable net benefit. In the independent test cohort, LR or BN was preferred at lower risk thresholds, whereas XGBoost offered greater net benefit at higher thresholds, consistent with their sensitivity- versus specificity-forward profiles. These results argue against selecting a model solely on discrimination and support aligning model choice with the intended decision threshold range [32].

### Limitations of Model Reliability

Calibration differentiated model reliability more clearly than discrimination [33]. In the development set, LR and BN were well calibrated, while XGBoost showed clear miscalibration consistent with overfitting. Calibration in held-out data was less stable and varied by evaluation cohort: in 1 test evaluation, LR retained acceptable calibration with mild underestimation at low predicted risks, whereas BN showed significant miscalibration; in the independent test cohort analysis with identical predictors, BN achieved the strongest overall calibration profile (despite underestimation at lower probabilities), LR was moderately miscalibrated, and XGBoost exhibited systematic deviation and unstable goodness-of-fit diagnostics due to sparse bins. Across these analyses, two points are consistent: (1) calibration is more fragile than discrimination and sensitive to dataset shift, and (2) XGBoost produced the least reliable probability estimates without recalibration, even when its point prediction error (Brier score) was competitive. Because risk stratification in practice relies on accurate probabilities rather than rankings alone, calibration performance should be a primary determinant of clinical readiness [34].

### Clinical Significance of Key Associated Factors

#### Core Associated Factors Identified Across Models

Across analytic approaches, psychiatric history was the strongest associated factor, with nephropathy, arthritis, and gastropathy consistently ranking among the most influential clinical correlates. Heart disease and hemoglobin also emerged as contributors in multivariable models, reinforcing the relevance of broader multimorbidity and systemic health in PDDC risk profiles [35]. The repeated identification of these conditions across LR, BN, and XGBoost interpretability analyses (OR/credible intervals and SHAP rankings) supports their robustness as markers of vulnerability.

Crucially, removing psychiatric history to address conceptual overlap with CESD-10 produced only modest declines in discrimination and preserved the associations for nephropathy, arthritis, gastropathy, household registration status, and BMI. This pattern indicates that the predictive framework was not dominated by psychiatric history alone and that the remaining variables contributed independent prognostic information. By contrast, several laboratory biomarkers that differed between groups at baseline (eg, uric acid, HbA_1c_, and platelet count) played a less consistent role in multivariable models, suggesting that their crude differences may be confounded or clinically modest in magnitude [36].

#### Impact of Sociodemographic Factors

Sociodemographic factors were consistently retained in the final models and were generally associated with lower PDDC risk. At baseline, rural household registration status and lower educational attainment were more common in the PDDC group; in adjusted analyses, both household registration status and education level were inversely associated with subsequent PDDC risk [37,38]. This pattern aligns with the influence of structural determinants—access to services, continuity of care, health literacy, and material constraints—on susceptibility to depressive symptoms among individuals with impaired pulmonary function [39,40].

The inverse association between BMI and PDDC risk contrasts with much of the Western literature, where obesity is often linked to higher depression risk. Several cohort- and context-specific considerations may account for this difference. In older Chinese adults, lower BMI more frequently reflects malnutrition, sarcopenia, or frailty, all of which are strongly associated with depressive symptoms. Moreover, an “obesity paradox” has been reported in Asian populations, whereby modestly higher BMI is associated with better outcomes in later life. In our cohort, BMI was relatively low overall (mean 22.9 kg/m²), and obesity was uncommon (BMI≥30 kg/m², approximately 4%), limiting power to detect nonlinear (U- or J-shaped) associations. Cultural norms may also shape the BMI-depression relationship by altering the social meaning of body weight and the extent of weight-related stigma [41,42]. Notably, BMI showed an inverse association across multiple models, consistent with the lower mean BMI observed in the PDDC group [43]; this likely captures vulnerability phenotypes—frailty, sarcopenia, or higher chronic disease burden—rather than a protective effect of lower weight per se [44]. Sex differences remained evident after adjustment in the unified analyses, suggesting heterogeneity in symptom reporting, comorbidity profiles, or access to care that merits further investigation.

#### Methodological Contributions and Innovations

This study contributes methodologically by evaluating 3 distinct modeling paradigms—linear (LR), probabilistic graphical (BN), and gradient-boosted trees (XGBoost)—under both model-specific and unified predictor settings. The evaluation emphasized not only discrimination but also calibration and DCA, aligning performance assessment with clinical decision-making rather than rank-based metrics alone. Interpretability was addressed through complementary tools (effect estimates for LR or BN and SHAP for XGBoost), enabling triangulation of core factors that remained consistent across methods. Finally, the sensitivity analysis excluding psychiatric history directly addressed a common concern in depression prediction and strengthened confidence that multimorbidity and sociodemographic variables provide independent predictive signal.

### Study Limitations

Several limitations should be considered. First, overall discrimination in external testing was modest, indicating that key determinants of PDDC risk remain unmeasured. Second, calibration was cohort dependent, and goodness-of-fit testing was unstable for XGBoost in the independent test cohort due to sparse bins, limiting inferential confidence from Hosmer-Lemeshow statistics. Third, CESD-10 is a screening tool rather than a diagnostic interview; outcome misclassification is possible. Fourth, comorbidity histories and behavioral measures may be subject to reporting or recording bias, and the cross-sectional design precludes causal interpretation. Fifth, our reliance on 2011 and 2015 CHARLS data, while necessary to obtain complete biomarker profiles, limits the temporal relevance of our findings. Health care accessibility, diagnostic practices, and population health characteristics may have evolved over the past decade. Finally, the feature set lacked clinically rich markers such as pulmonary function severity, symptom burden, medication exposure, and psychosocial stressors, which likely mediate or modify PDDC risk. Although the core biological and sociodemographic relationships we identified are likely to remain stable, validation with more recent cohort data is warranted when comprehensive biomarker panels become available.

### Future Research Directions

Future work should focus on (1) prospective, multisite external validation to quantify calibration drift and transportability; (2) formal recalibration and model updating, particularly for tree-based learners, before clinical use; (3) incorporation of disease severity measures and longitudinal trajectories to distinguish incident from persistent depressive symptoms; and (4) evaluation of clinically actionable thresholds in workflow-based impact studies to determine whether model-guided screening or referral improves outcomes. Given the contribution of sociodemographic factors, fairness and subgroup performance analyses should also be prioritized.

### Conclusions

Using routinely available clinical and sociodemographic data, LR and BN achieved discrimination comparable with XGBoost and, in several evaluations, provided more reliable probability estimates. Model selection should be driven by the intended clinical application: LR offers a balanced operating profile, BN is attractive when sensitivity is prioritized, and XGBoost may be considered for high-threshold decisions only with careful recalibration. Across modeling strategies, psychiatric history, nephropathy, arthritis, gastropathy, household registration status, and BMI emerged as stable associated factors, and the predictive signal persisted after excluding psychiatric history, underscoring the independent contribution of multimorbidity and social determinants to PDDC risk.

## Supplementary material

10.2196/77940 Multimedia Appendix 1 Variable coding, statistical analysis, and model performance of the study on pulmonary dysfunction–depression comorbidity.

## Abbreviations

AUROC: area under the receiver operating characteristic curve
BN: Bayesian network
CESD-10: 10-item Center for Epidemiologic Studies Depression Scale
CHARLS: China Health and Retirement Longitudinal Study
COPD: chronic obstructive pulmonary disease
DCA: decision curve analysis
FEV1%: forced expiratory volume in 1 second as a percentage of the predicted value
HbA_1c_: hemoglobin A_1c_
*ICD-10*: *International Statistical Classification of Diseases, Tenth Revision*
LASSO: Least Absolute Shrinkage and Selection Operator
LR: logistic regression
MCV: mean corpuscular volume
OR: odds ratio
PD: pulmonary dysfunction
PDDC: pulmonary dysfunction–depression comorbidity
SHAP: Shapley Additive Explanations
XGBoost: Extreme Gradient Boosting
