# Supplementary material for: Comparative Performance of 3 Analytical Models in Identifying Associated Factors of Pulmonary Dysfunction–Depression Comorbidity: China Health and Retirement Longitudinal Study–Based Nationwide Cross-Sectional Study
Source: JMIR Med Inform. 2026 Apr 9;14:e77940. doi: 10.2196/77940 (PMC13064592; doi:10.2196/77940)
Supplement: Multimedia Appendix 1 [file medinform-v14-e77940-s001.docx]

| **Table S1.** Coding of variables. | | |
| --- | --- | --- |
| Variable name | Variable definition | Attribute |
| **Demographic parameters** | | |
| Age | Participant's age in years | Continuous (year) |
| Sex | Biological sex | 1 =Male, 2 =Female |
| Body mass index(BMI) | Weight in kilograms divided by height in meters squared | Continuous (kg/m²) |
| Education levels | Highest educational attainment | 1 =primary school or below, 2 =middle school, 3 =high school or above |
| Household registration | Official residential registration type | 1 = rural, 2 = urban |
| Marry status | Legal partnership status | 1 =Married, 2 =Separated and divorced, 3 =Widowed, 4 =Never married |
| **Blood-based biomarkers** | | |
| Glucose | Fasting plasma glucose concentration | Continuous (mg/dl) |
| Cholesterol | Total serum cholesterol level | Continuous (mg/dl) |
| High-density lipoprotein (HDL) | Serum HDL cholesterol concentration | Continuous (mg/dl) |
| Low-density lipoprotein (LDL) | Serum LDL cholesterol concentration | Continuous (mg/dl) |
| Triglycerides (TG) | Serum triglyceride level | Continuous (mg/dl) |
| Blood urea nitrogen (BUN) | Serum urea nitrogen concentration | Continuous (mg/dl) |
| Creatine | Serum creatinine level reflecting muscle metabolism | Continuous (mg/dl) |
| Uric acid | Serum uric acid concentration | Continuous (mg/dl) |
| High-sensitivity C-reactive protein (hsCRP) | Ultra-sensitive assay of systemic inflammation marker | Continuous (mg/dl) |
| Glycosylated hemoglobin (Hba1c) | Percentage of glycated hemoglobin reflecting 3-month glucose control | Continuous (%) |
| Hematocrit | Volume percentage of red blood cells in blood | Continuous (mg/dl) |
| Hemoglobin | Blood hemoglobin concentration | Continuous (mg/dl) |
| White blood cell (WBC) | Total leukocyte count in peripheral blood | Continuous (10^9^/L) |
| Mean corpuscular volume (MCV) | Average volume of red blood cells | Continuous (fl) |
| Platelet | Thrombocyte count in peripheral blood | Continuous (10^9^/L) |
| **Chronic disease comorbidity** | | |
| Hypertension | Self-reported or physician-diagnosed high blood pressure (≥140/90 mmHg or on antihypertensives) | 0 = No, 1 = Yes |
| Diabetes | Self-reported or physician-diagnosed diabetes (fasting glucose ≥7.0mmol/L or HbA1c ≥6.5%) | 0 = No, 1 = Yes |
| Cancer | "Have you been diagnosed with cancer (excluding skin cancer)?" | 0 = No, 1 = Yes |
| Hyperlipidemia | Self-reported diagnosis OR meeting any of the following lab criteria: HDL < 35 mg/dL ,LDL > 130 mg/dL,Total cholesterol > 200 mg/dL,Triglycerides > 150 mg/dL | 0 = No, 1 = Yes |
| Hepatopathy | "Have you been diagnosed with chronic liver disease?" | 0 = No, 1 = Yes |
| Heart diseases | "Have you been diagnosed with heart disease (angina, heart attack, etc.)?" | 0 = No, 1 = Yes |
| Stroke | "Have you been diagnosed with a stroke?" | 0 = No, 1 = Yes |
| Nephropathy | "Have you been diagnosed with chronic kidney disease?" | 0 = No, 1 = Yes |
| Gastropathy | "Have you been diagnosed with chronic stomach disease or ulcer?" | 0 = No, 1 = Yes |
| Mental illness | "Have you been diagnosed with depression/anxiety/other mental illness?" | 0 = No, 1 = Yes |
| Memory-associated disorders | "Have you been diagnosed with dementia or memory problems?" | 0 = No, 1 = Yes |
| Arthritis | "Have you been diagnosed with arthritis?" | 0 = No, 1 = Yes |
| Asthma | "Have you been diagnosed with asthma?" | 0 = No, 1 = Yes |
| **Behavioral determinants** | | |
| Smoking | “Have you ever chewed tobacco, smoked a pipe, smoked self-rolled cigarettes, or smoked cigarettes/cigars?” | 0 =Non-smoker  1 =Smoker |
| Drinking | “Did you drink any alcoholic beverages, such as beer, wine, or liquor in the past year? How often？” | 1 = Drink more than once a month  2 =Drink but less than once a month  3 = None of these |

**Table S2.** All analyses were performed in R v4.4.3 using validated computational libraries.

**The corresponding run codes are provided:**

install.packages("randomForest")

library(randomForest)

data$Dispression <- as.factor(data$Dispression)

data <- data[, !colnames(data) %in% "ID"]

set.seed(123)

train_index <- sample(1:nrow(data), 0.8 * nrow(data))

train_data_final <- data[train_index, ]

test_data <- data[-train_index, ]

rf_model <- randomForest(Dispression ~ ., data = data, importance = TRUE)

print(rf_model)

predictions <- predict(rf_model, newdata = test_data)

predicted_prob1 <- predict(rf_model, newdata = test_data, type = "prob")[, 2]

importance(rf_model)

varImpPlot(rf_model, main = "Variable Importance")

varImpPlot(rf_model,

main = "Variable Importance")

library(brms)

library(rstan)

data<-read.csv("C:/Users/admin/Desktop/抑郁-结核共病研究/shujuzongjie.csv")

str(data)

library(dplyr)

data <- data %>%

mutate_if(is.integer, as.factor)

numeric_cols <- sapply(data, is.numeric)

data[numeric_cols] <- scale(data[numeric_cols])

summary(data[numeric_cols])

summary(fit1)

library(caret)

set.seed(42)

trainIndex <- createDataPartition(data$Depression, p = 0.8, list = FALSE)

trainData <- data[trainIndex, ]

testData <- data[-trainIndex, ]

x_train <- model.matrix(Depression ~ ., data = train_data)[,-1]

y_train <- train_data$Despression

fit1 <- brm( formula = Depression ~ Arthritis+Gastropathy+Education+Gender+LDL+Cancer+Mental_illness+Household_registration+MCV+Nephropathy+Drink+BMI+Uricacid+Hemoglobin+Age+Stroke+Hematocrit+Creatine+Hba1c+Heart_Disease,

data = train_data,

family = bernoulli(),

chains = 4, iter = 2000, warmup = 1000, cores = 4

)

fit2 <- glm(Depression ~ Creatine+Uricacid+Hba1c+MCV+Platelet+Drink+Gender+Household_registration+Education+BMI+Hyperlipidemia+Cancer+Heart_Disease+Nephropathy+Gastropathy+Mental_illness+Arthritis, data = train_data, family = binomial)

summary(fit1)

library(caret)

x_test <- model.matrix(dispression ~ ., data = testData)[,-1]

y_test <- testData$dispression

lambda_grid <- 10^seq(-4, 1, length = 100)

set.seed(42)

lasso_cv <- cv.glmnet(

x = x_train,

y = y_train,

alpha = 1,

family = "binomial",

lambda = lambda_grid,

type.measure = "deviance",

nfolds = 5,

standardize = TRUE

)

best_lambda <- lasso_cv$lambda.min

print(paste("Best lambda:", best_lambda))

plot(lasso_cv)

lasso_model <- glmnet(

x = x_train,

y = y_train,

alpha = 1,

family = "binomial",

lambda = best_lambda,

standardize = TRUE

)

lasso_coef <- coef(lasso_model, s = best_lambda)

selected_features <- rownames(lasso_coef)[which(lasso_coef != 0)][-1]

print("Selected features:")

print(selected_features)

print("Coefficients of selected features:")

print(lasso_coef[which(lasso_coef != 0), ])

lasso_full <- glmnet(

x = x_train,

y = y_train,

alpha = 1,

family = "binomial",

lambda = lambda_grid,

standardize = TRUE

)

plot(lasso_full, xvar = "lambda", label = TRUE)

abline(v = log(best_lambda), lty = 2)

coef_df <- data.frame(

feature = rownames(lasso_coef)[-1],

coefficient = as.vector(lasso_coef[-1, ])

) %>%

filter(coefficient != 0) %>%

arrange(desc(abs(coefficient)))

ggplot(coef_df, aes(x = reorder(feature, coefficient), y = coefficient, fill = coefficient > 0)) +

geom_bar(stat = "identity") +

coord_flip() +

scale_fill_manual(values = c("red", "blue"), name = "Direction", labels = c("Negative", "Positive")) +

labs(title = "LASSO Selected Features and Coefficients",

x = "Feature",

y = "Coefficient Value") +

theme_minimal()

lasso_model_1se <- glmnet(

x = x_train,

y = y_train,

alpha = 1,

family = "binomial",

lambda = lambda_1se,

standardize = TRUE

)

coef_1se <- coef(lasso_model_1se)

selected_features_1se <- rownames(coef_1se)[which(coef_1se != 0)][-1]

print(paste("Number of features selected:", length(selected_features_1se)))

coef_1se <- coef(lasso_model_1se, s = lambda_1se)

selected_vars <- rownames(coef_1se)[which(coef_1se != 0)][-1] # 移除截距项

print(paste("Selected variables (n =", length(selected_vars), "):"))

print(selected_vars)

library(xgboost)

library(caret)

data$dispression <- as.numeric(data$dispression) - 1

set.seed(42)

trainIndex <- createDataPartition(data$dispression, p = 0.8, list = FALSE)

trainData <- data[trainIndex, c("dispression", selected_vars)]

testData <- data[-trainIndex, c("dispression", selected_vars)]

x_train <- as.matrix(trainData[, selected_vars])

y_train <- trainData$dispression

x_test <- as.matrix(testData[, selected_vars])

y_test <- testData$dispression

dtrain <- xgb.DMatrix(data = x_train, label = y_train)

dtest <- xgb.DMatrix(data = x_test, label = y_test)

params <- list(

objective = "binary:logistic",

eval_metric = "auc",

eta = 0.1,

max_depth = 6,

subsample = 0.8,

colsample_bytree = 0.8,

gamma = 0.1,

min_child_weight = 1,

scale_pos_weight = sum(y_train == 0) / sum(y_train == 1)

)

set.seed(42)

fit3 <- xgb.train(

params = params,

data = dtrain,

nrounds = 500,

watchlist = list(train = dtrain, test = dtest),

early_stopping_rounds = 50,

print_every_n = 20,

verbose = 1

)

set.seed(42)

trainIndex <- createDataPartition(data$dispression, p = 0.7, list = FALSE)

trainData <- data[trainIndex, ]

testData <- data[-trainIndex, ]

if(class(fit1) == "glm"){

prob_fit1 <- predict(fit1, newdata = data, type = "response")

} else {

prob_fit1 <- predict(fit1, newdata = test_data, type = "prob")[,2]

}

prob_fit1 <- as.numeric(prob_fit1)

prob_fit22 <- predict(fit2, newdata = test_data, type = "prob")[,2]

prob_fit2 <- predict(fit2, newdata = test_data, type = "response")

test_matrix <- model.matrix(dispression ~ . -1, data = test_data)

dtest <- xgb.DMatrix(data = test_matrix, label = test_data$dispression)

prob_fit3 <- predict(fit3, dtest)

library(pROC)

roc_fit1 <- roc(data$dispression, prob_fit1)

roc_fit2 <- roc(data$dispression, prob_fit2)

roc_fit3 <- roc(data$dispression, prob_fit3)

auc_fit1 <- round(auc(roc_fit1), 4)

auc_fit2 <- round(auc(roc_fit2), 4)

auc_fit3 <- round(auc(roc_fit3), 4)

ggroc_obj <- ggroc(list(

BayesNet = roc_fit1,

Logistic = roc_fit2,

XGBoost = roc_fit3

), legacy.axes = TRUE) +

geom_segment(aes(x = 0, xend = 1, y = 0, yend = 1),

color = "grey", linetype = "dashed") +

theme_minimal() +

labs(x = "False Positive Rate (1 - Specificity)",

y = "True Positive Rate (Sensitivity)",

title = "ROC Curve Comparison") +

scale_color_manual(

name = "Models (AUC)",

values = c("Logistic" = "#E41A1C",

"BayesNet" = "#377EB8",

"XGBoost" = "#4DAF4A"),

labels = c(

paste0("Logistic Regression (",auc_fit2 , ")"),

paste0("Bayesian Network (",auc_fit1 , ")"),

paste0("XGBoost (", auc_fit3, ")")

)

) +

theme(

legend.position = c(0.7, 0.2),

legend.background = element_rect(fill = "white", color = "black"),

plot.title = element_text(hjust = 0.5, face = "bold"),

panel.grid.major = element_line(color = "gray90"),

panel.grid.minor = element_blank()

)

print(ggroc_obj)

data_sens <- data[data$Mental_illness != 1, ]

vars <- c("Depression", "Household_registration", "Gender", "Education", "BMI", "Marry",

"Creatine", "Hba1c", "MCV", "Platelet", "LDL", "Uricacid",

"Hemoglobin", "Cancer", "Nephropathy", "Heart_Disease",

"Gastropathy", "Arthritis", "Stroke", "Drink")

vars <- vars[vars %in% names(data_sens)]

df <- data_sens[, vars]

set.seed(123)

train_idx <- sample(1:nrow(df), 0.8 * nrow(df))

train <- df[train_idx, ]

test <- df[-train_idx, ]

bayes_formula <- as.formula(paste("Depression ~", paste(setdiff(names(train), "Depression"), collapse = " + ")))

bayes_model <- brm(

formula = bayes_formula,

data = train,

family = bernoulli(),

chains = 2,

iter = 1000,

warmup = 500,

cores = 2,

seed = 123,

refresh = 0

)

logistic_model <- glm(bayes_formula, data = train, family = binomial())

xgb_train <- train

xgb_test <- test

factor_cols <- sapply(xgb_train, is.factor)

for(col in names(factor_cols)[factor_cols]) {

if(col != "Depression") {

xgb_train[[col]] <- as.numeric(xgb_train[[col]]) - 1

xgb_test[[col]] <- as.numeric(xgb_test[[col]]) - 1

}

}

features <- setdiff(names(xgb_train), "Depression")

train_matrix <- as.matrix(xgb_train[, features])

test_matrix <- as.matrix(xgb_test[, features])

dtrain <- xgb.DMatrix(data = train_matrix, label = train$Depression)

dtest <- xgb.DMatrix(data = test_matrix, label = test$Depression)

xgb_model <- xgb.train(

data = dtrain,

nrounds = 100,

objective = "binary:logistic",

eval_metric = "auc",

max_depth = 4,

eta = 0.1,

verbose = 0

)

bayes_train_pred <- predict(bayes_model, newdata = train, type = "response")[, "Estimate"]

bayes_test_pred <- predict(bayes_model, newdata = test, type = "response")[, "Estimate"]

logistic_train_pred <- predict(logistic_model, newdata = train, type = "response")

logistic_test_pred <- predict(logistic_model, newdata = test, type = "response")

xgb_train_pred <- predict(xgb_model, dtrain)

xgb_test_pred <- predict(xgb_model, dtest)

auc_bayes_train <- auc(train$Depression, bayes_train_pred)

auc_bayes_test <- auc(test$Depression, bayes_test_pred)

auc_logistic_train <- auc(train$Depression, logistic_train_pred)

auc_logistic_test <- auc(test$Depression, logistic_test_pred)

auc_xgb_train <- auc(train$Depression, xgb_train_pred)

auc_xgb_test <- auc(test$Depression, xgb_test_pred)

roc_bayes_train <- roc(train$Depression, bayes_train_pred, quiet = TRUE)

roc_bayes_test <- roc(test$Depression, bayes_test_pred, quiet = TRUE)

roc_logistic_train <- roc(train$Depression, logistic_train_pred, quiet = TRUE)

roc_logistic_test <- roc(test$Depression, logistic_test_pred, quiet = TRUE)

roc_xgb_train <- roc(train$Depression, xgb_train_pred, quiet = TRUE)

roc_xgb_test <- roc(test$Depression, xgb_test_pred, quiet = TRUE)

par(mfrow = c(1, 2))

plot(roc_bayes_train, col = "red", main = "Training Set ROC Curves")

lines(roc_logistic_train, col = "blue")

lines(roc_xgb_train, col = "green")

legend("bottomright", legend = c("Bayesian", "Logistic", "XGBoost"),

col = c("red", "blue", "green"), lwd = 2)

plot(roc_bayes_test, col = "red", main = "Test Set ROC Curves")

lines(roc_logistic_test, col = "blue")

lines(roc_xgb_test, col = "green")

legend("bottomright", legend = c("Bayesian", "Logistic", "XGBoost"),

col = c("red", "blue", "green"), lwd = 2)

bayes_samples <- as_draws_df(bayes_model)

coef_cols <- grep("^b_", colnames(bayes_samples), value = TRUE)

coef_cols <- coef_cols[coef_cols != "b_Intercept"]

bayes_or_summary <- data.frame(

Variable = gsub("^b_", "", coef_cols),

OR = apply(exp(bayes_samples[, coef_cols]), 2, median),

CI_2.5 = apply(exp(bayes_samples[, coef_cols]), 2, quantile, probs = 0.025),

CI_97.5 = apply(exp(bayes_samples[, coef_cols]), 2, quantile, probs = 0.975)

)

logistic_or <- exp(cbind(OR = coef(logistic_model), confint.default(logistic_model)))

logistic_or_summary <- data.frame(

Variable = rownames(logistic_or),

OR = logistic_or[, "OR"],

CI_2.5 = logistic_or[, "2.5 %"],

CI_97.5 = logistic_or[, "97.5 %"]

)

shap_values <- shap.values(xgb_model = xgb_model, X_train = train_matrix)

shap_importance <- shap_values$mean_shap_score

shap_importance_df <- data.frame(

Feature = names(shap_importance),

SHAP_Importance = as.numeric(shap_importance)

)

| **Table S3.** Baseline characteristics of the study population. | | | |
| --- | --- | --- | --- |
| Variables^a,b^ | Group no-PDDC  (N= 632) | Group PDDC  (N =514) | *P* value |
| **Demographic parameters**  Age, years [Mean (SD)] | 62.48 (9.93) | 62.16 (9.67) | .58 |
| Body mass index, kg/m²[Mean (SD)] | 23.20 (4.22) | 22.54 (3.71) | .01 |
| Sex, n (%) |  |  | <.001 |
| Male | 372 (58.86) | 232 (45.14) |  |
| Female | 260 (41.14) | 282 (54.86) |  |
| Education level, n (%) |  |  | <.001 |
| Primary school or below | 368（58.23） | 394（76.66） |  |
| Middle school | 95（15.03） | 55（10.70） |  |
| High school or above | 169（26.74) | 65（12.64） |  |
| Household registration, n (%) |  |  | <.001 |
| Rural area | 417(65.99) | 414 (80.54) |  |
| Urban area | 215(34.01) | 100 (19.46) |  |
| Marry status, n (%) |  |  | .08 |
| Married | 548 (87.26) | 422 (81.78) |  |
| Separated and divorced | 3 ( 0.48) | 7 ( 1.36) |  |
| Widowed | 77 (12.26) | 78 (15.12) |  |
| Never married | 4 ( 0.64) | 7 ( 1.36) |  |
| **Blood-based biomarkers, Mean (SD)** |  |  |  |
| Glucose, mg/dL | 106.07(31.12) | 104.41(23.14) | .32 |
| Cholesterol, mg/dL | 190.41(37.70) | 189.91(36.57) | .82 |
| HDL, mg/dL | 53.19(16.27) | 53.45(15.52) | .79 |
| LDL, g/dL | 111.80(32.88) | 113.04(30.34) | .81 |
| TG, mg/dL | 102.66(84.00) | 102.66(67.00) | .82 |
| BUN, mg/dL | 15.98(4.81) | 15.84(4.89) | .14 |
| Creatine, mg/dL | 0.83(0.24) | 0.78(0.21) | .05 |
| Uricacid, mg/dL | 4.81(1.37) | 4.43(1.20) | <.001 |
| hsCRP, mg/dL | 1.22(2.00) | 1.20(2.00) | .30 |
| Hba1c, % | 5.30(1.00) | 5.20(1.00) | 0.002 |
| Hematocrit, mg/dL | 42.01(5.89) | 41.52(5.93) | .16 |
| Hemoglobin, mg/dL | 14.35(2.02) | 14.41(2.18) | .66 |
| WBC, 10^9^/L | 5.90(2.00) | 6.00(3.00) | .34 |
| MCV, fl | 91.59(8.47) | 90.25(8.91) | .01 |
| Platelet,10^9^/L | 194.00(95.00) | 202.00(93.00) | .05 |
| **Chronic disease comorbidity, n (%)** | | | |
| Hypertension |  |  | .90 |
| No | 413 (65.35) | 338 (65.76) |  |
| Yes | 219 (34.65) | 176 (34.24) |  |
| Diabets |  |  | .40 |
| No | 547 (86.55) | 435 (84.63) |  |
| Yes | 85 (13.45) | 79 (15.37) |  |
| Cancer |  |  | .02 |
| No | 629 (99.52) | 504 (98.05) |  |
| Yes | 3 (0.48) | 10 (1.95) |  |
| Hyperlipidemia |  |  | .001 |
| No | 351 (55.54) | 233 (45.33) |  |
| Yes | 281 (44.46) | 281 (54.67) |  |
| Hepatopathy |  |  | .36 |
| No | 591 (93.51) | 473 (92.02) |  |
| Yes | 41 (6.49) | 41 (7.98) |  |
| Heart disease |  |  | <.001 |
| No | 525 (83.06) | 364 (70.82) |  |
| Yes | 107 (16.94) | 150 (29.18) |  |
| Stroke |  |  | .10 |
| No | 620 (98.10) | 496 (96.50) |  |
| Yes | 12 (1.90) | 18 (3.50) |  |
| Nephropathy |  |  | <.001 |
| No | 586 (92.72) | 426 (82.87) |  |
| Yes | 46 (7.28) | 88 (17.13) |  |
| Gastropathy |  |  | <.001 |
| No | 502 (79.43) | 303 (58.95) |  |
| Yes | 130 (20.57) | 211 (41.05) |  |
| Mental illness |  |  | <.001 |
| No | 626 (99.05) | 493 (95.91) |  |
| Yes | 6 (0.95) | 21 (4.09) |  |
| Memory-associated disorders |  |  | .43 |
| No | 620 (98.10) | 500 (97.28) |  |
| Yes | 12 (1.90) | 14 (2.72) |  |
| Arthritis |  |  | <.001 |
| No | 425 (67.25) | 233 (45.33) |  |
| Yes | 207 (32.75) | 281 (54.67) |  |
| Asthma |  |  | .09 |
| No | 520 (82.28) | 402 (78.21) |  |
| Yes | 112 (17.72) | 112 (21.79) |  |
| **Behavioral determinants, n (%)** |  |  |  |
| Smoking |  |  | .55 |
| Non-smoker | 280 (44.30) | 218 (42.41) |  |
| Smoker | 352 (55.70) | 296 (57.59) |  |
| Drinking |  |  | <.001 |
| Drink more than once a month | 171 (27.11) | 98 (19.06) |  |
| Drink but less than once a month | 56 (8.88) | 30 (5.83) |  |
| None of these | 405 (64.01) | 386 (75.10) |  |
| ᵃ Continuous variables are presented as mean ± standard deviation. Normality was assessed using the Shapiro-Wilk test. Variables meeting normality assumptions (BMI, glucose, HDL, LDL, MCV, hemoglobin) were compared using Student's t-test. Non-normally distributed variables (TG, BUN, creatinine, uric acid, hsCRP, HbA1c, hematocrit, WBC, platelet) were compared using the Mann-Whitney U test.  ᵇ Categorical variables are presented as n (%) and compared using Pearson's chi-square test or Fisher's exact test (for expected cell counts < 5).  Abbreviations: BMI: body mass index; PDDC: pulmonary dysfunction-depression comorbidity; SD: standard deviation; HDL: high-density lipoprotein; LDL: low-density lipoprotein; TG: triglycerides; BUN: blood urea nitrogen; hsCRP: high-sensitivity C-reactive protein; HbA1c: glycosylated hemoglobin; WBC: white blood cell; MCV: mean corpuscular volume. | | | |

**Figure S1.** Visualization of variable importance measures derived from random forest algorithm in the non-unified feature set. Abbreviations: HDL: high-density lipoprotein; HR: household registration; MAD: memory-associated disorders; LDL: low-density lipoprotein; BMI: body mass index; TG: triglycerides; BUN: blood urea nitrogen; hsCRP: high-sensitivity C-reactive protein; HbA1c: glycosylated hemoglobin; WBC: white blood cell; MCV: mean corpuscular volume.

**
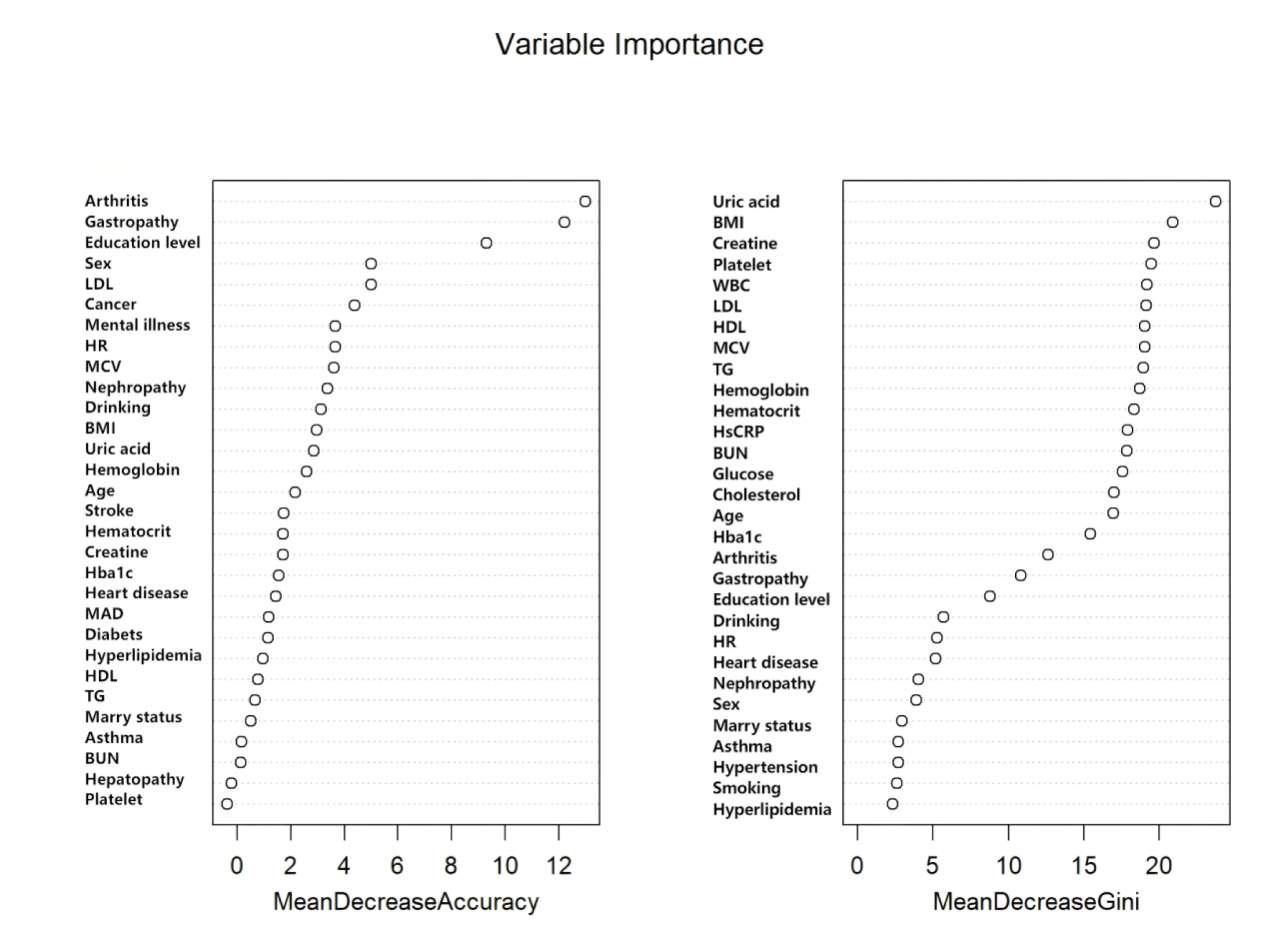
**

**Figure S2.** Visualization of variable importance/coefficients selected by Least Absolute Shrinkage and Selection Operator regression in the non-unified feature set..

Abbreviations: MCV: mean corpuscular volume; BMI: body mass index.

**
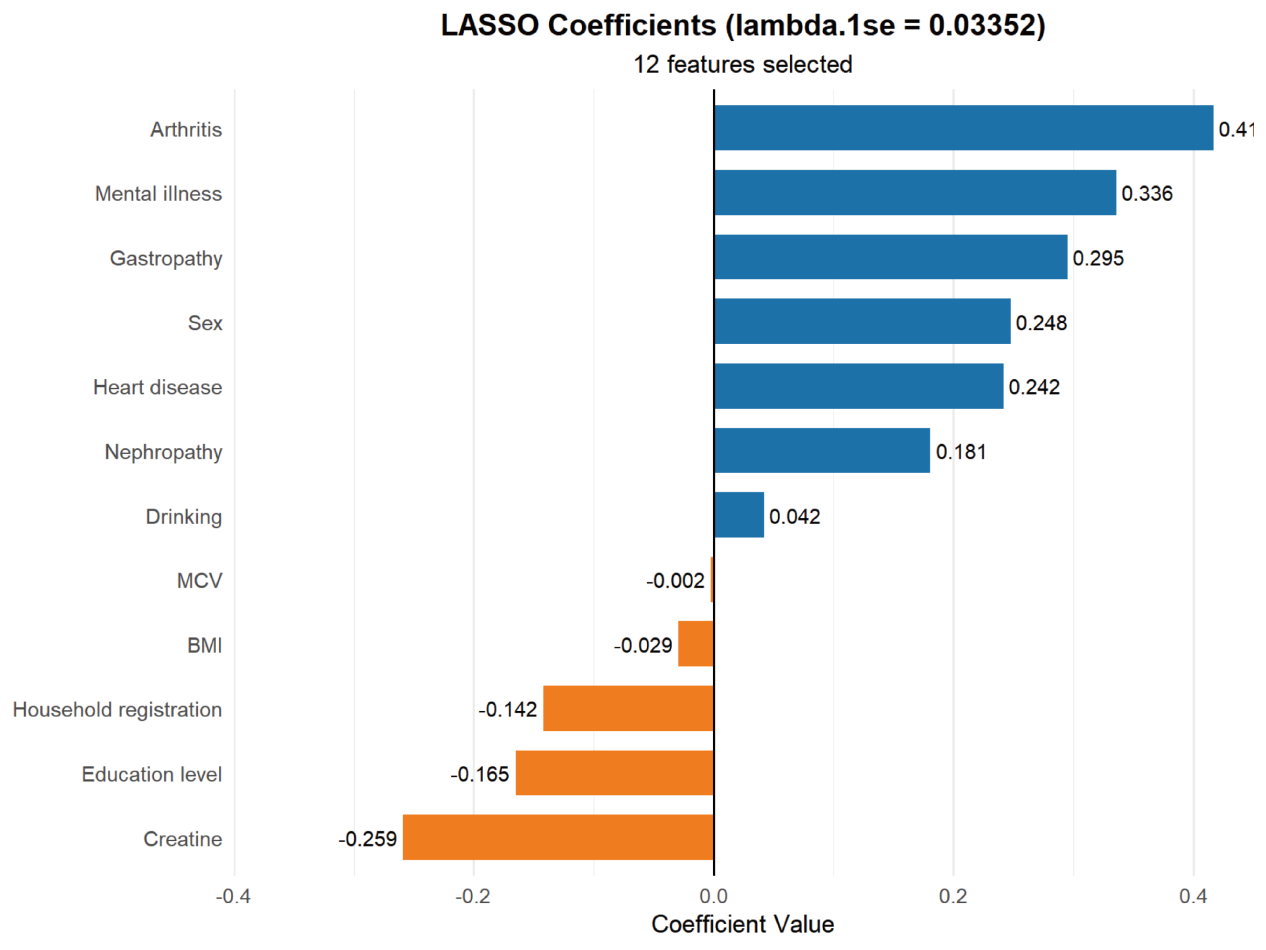
**

**Table S4.** Delong Test for the area under the receiver operating characteristic curve of three models in the non-unified feature set ^a^.

|  | eXtreme Gradient Boosting Model | Multivariable Logistic Regression Model | Bayesian Network Model |
| --- | --- | --- | --- |
| eXtreme Gradient Boosting Model | - |  |  |
| Multivariable Logistic Regression Model | <0.05^b^, 0.691^c^ | - |  |
| Bayesian Network Model | <0.05^b^, 0.875^c^ | 0.870^b^, 0.373^c^ | - |

^a^ All models were trained on the same feature set after standardization.

^b^ Non-unified feature set analysis.

^c^ Unified feature set analysis.

**Figure S3.** Visualization analyses of the eXtreme Gradient Boosting model in the unified feature set.

Abbreviations: LDL: low-density lipoprotein; BMI: body mass index; Hba1c: glycosylated hemoglobin; MCV: mean corpuscular volume; SHAP: Shapley Additive exPlanations.


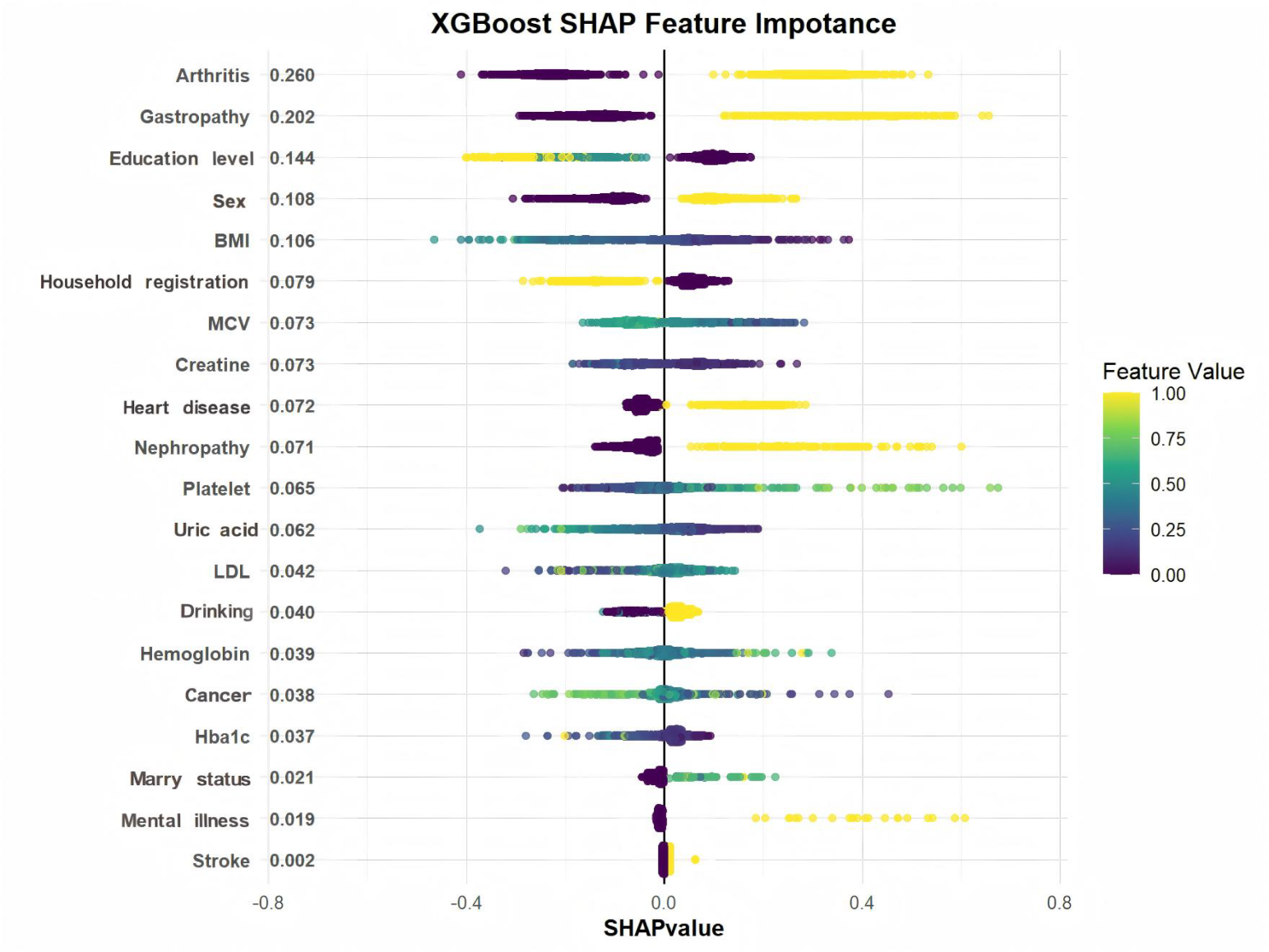


| Variables | **Logistic model** |  | **Bayesian model** |  | **XGboost model** |
| --- | --- | --- | --- | --- | --- |
| Training set AUC | 0.741 |  | 0.736 |  | 0.812 |
| Test set AUC | 0.651 |  | 0.662 |  | 0.637 |
|  | Odds ratios (95% confidence intervals) |  | Odds ratios (95% confidence intervals) |  | SHAP |
| Household registration | 0.68 (0.47–0.99) |  | 0.67 (0.47–1.01) |  | 0.323 |
| Sex | 1.76 (1.21–2.58) |  | 1.78 (1.23–2.58) |  | 0.270 |
| Education level | 0.82 (0.66–1.02) |  | 0.82 (0.64–1.02) |  | 0.261 |
| BMI | 0.92 (0.88–0.96) |  | 0.92 (0.88–0.95) |  | 0.234 |
| Marry status | 1.26 (1.03–1.56) |  | 1.27 (1.05–1.60) |  | 0.201 |
| Creatine | 0.84 (0.35–1.98) |  | 0.79 (0.35–2.04) |  | 0.182 |
| Hba1c | 0.90 (0.74–1.09) |  | 0.89 (0.72–1.07) |  | 0.145 |
| MCV | 0.98 (0.96–0.99) |  | 0.98 (0.96–0.99) |  | 0.139 |
| Platelet | 1.00 (1.00–1.00) |  | 1.00 (1.00–1.00) |  | 0.136 |
| LDL | 1.00 (1.00–1.01) |  | 1.00 (1.00–1.01) |  | 0.120 |
| Uricacid | 0.99 (0.86–1.13) |  | 0.99 (0.87–1.13) |  | 0.112 |
| Hemoglobin | 1.11 (1.03–1.20) |  | 1.12 (1.04–1.21) |  | 0.100 |
| Cancer | 4.01 (0.77–20.91) |  | 4.39 (0.92–36.43) |  | 0.086 |
| Nephropathy | 1.93 (1.19–3.13) |  | 2.00 (1.25–3.18) |  | 0.076 |
| Heart Disease | 1.26 (0.87–1.81) |  | 1.24 (0.91–1.79) |  | 0.067 |
| Gastropathy | 1.92 (1.37–2.68) |  | 1.94 (1.37–2.80) |  | 0.063 |
| Arthritis | 1.85 (1.35–2.53) |  | 1.89 (1.36–2.60) |  | 0.060 |
| Stroke | 1.63 (0.63–4.24) |  | 1.72 (0.59–4.62) |  | 0.002 |
| Drinking | 1.15 (0.95–1.39) |  | 1.15 (0.96–1.40) |  | 0.001 |
| Abbreviations: BMI: body mass index; LDL: low-density lipoprotein; Hba1c: glycosylated hemoglobin; MCV: mean corpuscular volume; SHAP: Shapley Additive Explanations; AUC: Area Under the Receiver Operating Characteristic Curve. | | | | | |

**Table S5.** Based on a consistent set of variables after excluding individuals with psychiatric disorders, this table presents the results from three models: odds ratios for the logistic and Bayesian models, and SHAP values for the XGBoost model.

**Figure S4.** Comparative performance of the three models in analyzing pulmonary dysfunction-depression comorbidity through receiver operating characteristic (ROC) analysis in the unified feature set. (A) Discriminative capacity of logistic regression, Bayesian network, and eXtreme Gradient Boosting models in the training dataset. (B) External validation of model generalizability in the test set.


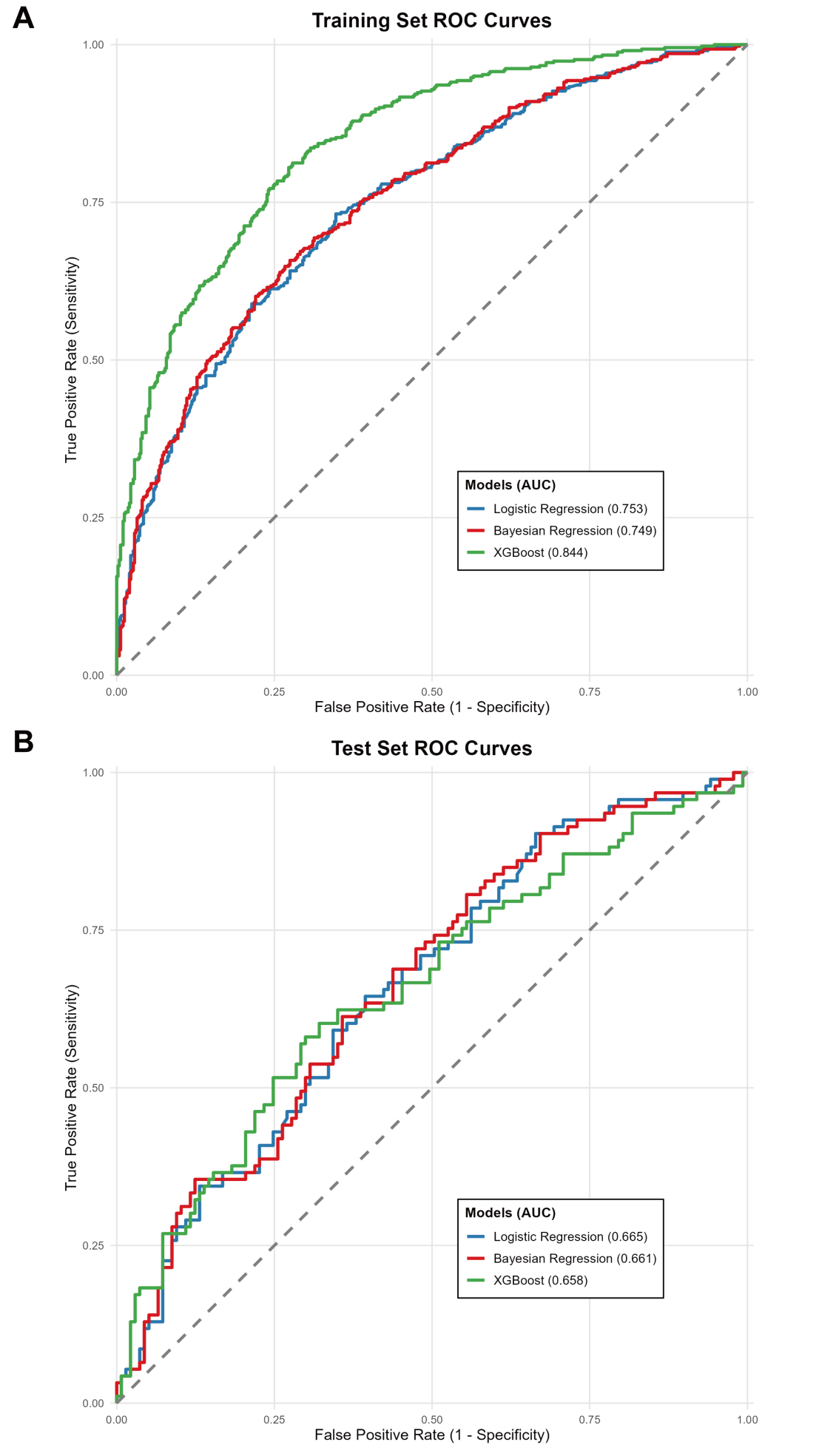


**Figure S5.** Calibration curve analyses for logistic regression, Bayesian network, and eXtreme Gradient Boosting models in analyzing pulmonary dysfunction-depression comorbidity in the unified feature set. (A) Training set. (B) Test set.

**
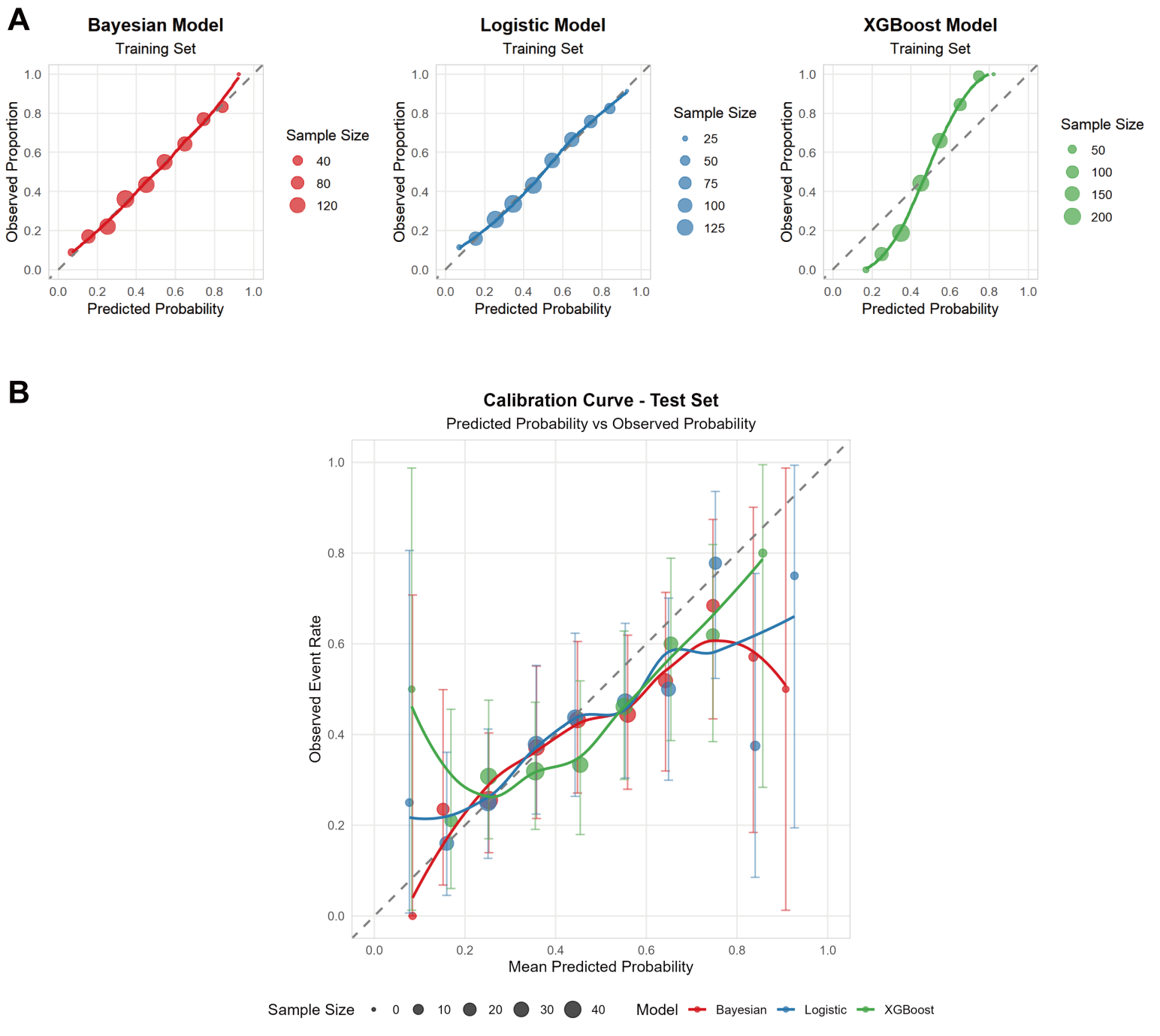
**

**Figure S6.** Decision curve analyses for logistic regression, Bayesian network, and eXtreme Gradient Boosting models in analyzing pulmonary dysfunction-depression comorbidity in the unified feature set. (A) Training set. (B) Test set.

**
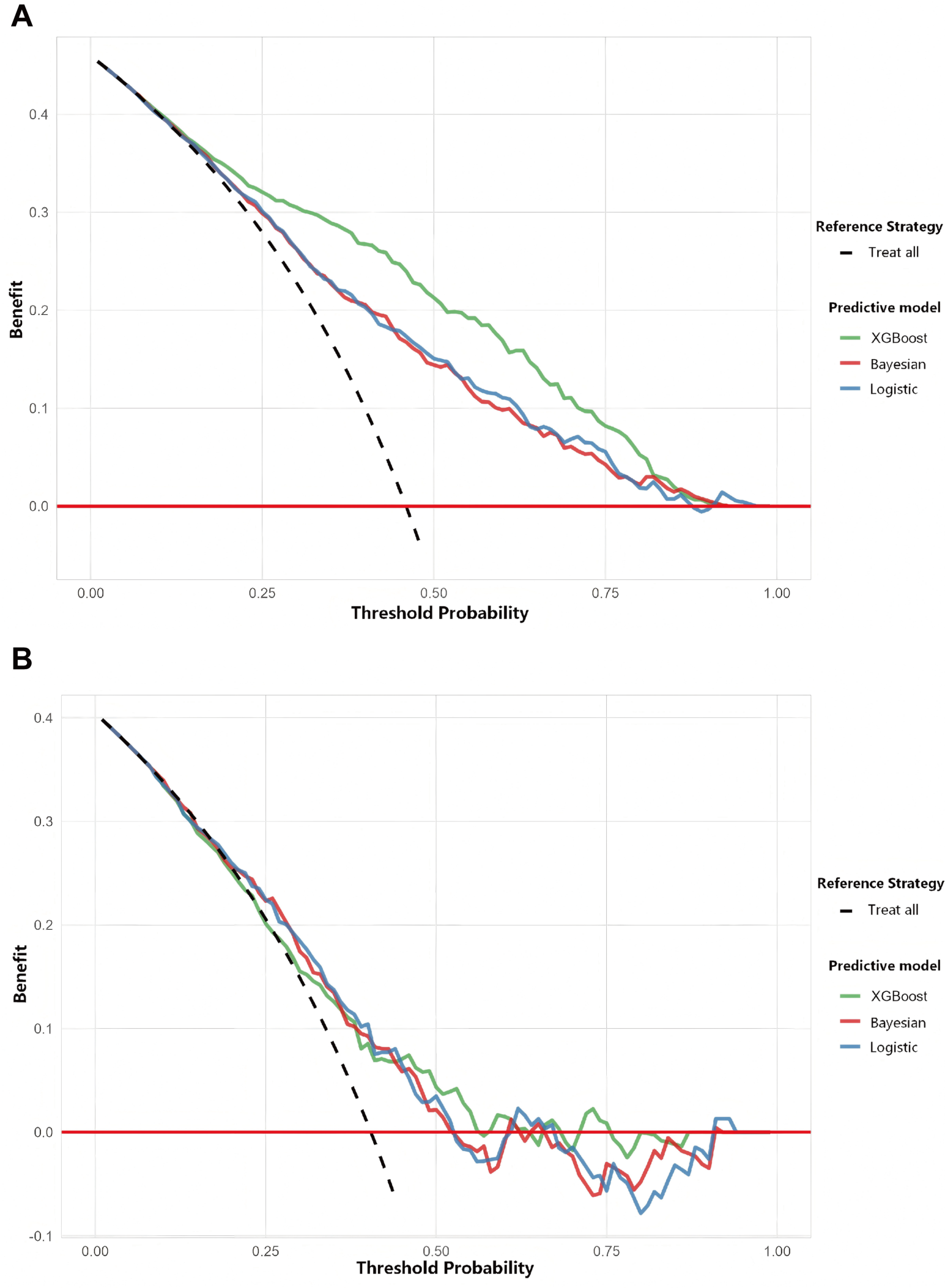
**
